# Identification of Climate and Genetic Factors That Control Fat Content and Fatty Acid Composition of *Theobroma cacao* L. Beans

**DOI:** 10.3389/fpls.2019.01159

**Published:** 2019-10-14

**Authors:** Guiliana M. Mustiga, Joe Morrissey, Joseph Conrad Stack, Ashley DuVal, Stefan Royaert, Johannes Jansen, Carolina Bizzotto, Cristiano Villela-Dias, Linkai Mei, Edgar B. Cahoon, Ed Seguine, Jean Philippe Marelli, Juan Carlos Motamayor

**Affiliations:** ^1^Mars Chocolate, Miami, FL, United States; ^2^University of Wageningen, Wageningen, Netherlands; ^3^Plant Sciences, Mars Center for Cocoa Science, Itajuípe, Brazil; ^4^Department of Biochemistry and Center for Plant Science Innovation, University of Nebraska-Lincoln, NE, United States; ^5^Seguine Cacao/Guittard Chocolate Co, Arroyo Grande, CA, United States

**Keywords:** cacao, fat content, fatty acid composition, weather, QTL, SNP, linkage mapping, heritability

## Abstract

The main ingredients of chocolate are usually cocoa powder, cocoa butter, and sugar. Both the powder and the butter are extracted from the beans of the cacao tree (*Theobroma cacao* L.). The cocoa butter represents the fat in the beans and possesses a unique fatty acid profile that results in chocolate’s characteristic texture and mouthfeel. Here, we used a linkage mapping population and phenotypic data of 3,292 samples from 420 progeny which led to the identification of 27 quantitative trait loci (QTLs) for fatty acid composition and six QTLs for fat content. Progeny showed extensive variation in fat levels and composition, with the level of palmitic acid negatively correlated to the sum of stearic acid, oleic acid, and linoleic acid. A major QTL explaining 24% of the relative level of palmitic acid was mapped to the distal end of chromosome 4, and those higher levels of palmitic acid were associated with the presence of a haplotype from the “TSH 1188” parent in the progeny. Within this region of chromosome 4 is the *Thecc1EG017405* gene, an orthologue and isoform of the stearoyl-acyl carrier protein (ACP) desaturase (SAD) gene in plants, which is involved in fatty acid biosynthesis. Besides allelic differences, we also show that climate factors can change the fatty acid composition in the beans, including a significant positive correlation between higher temperatures and the higher level of palmitic acid. Moreover, we found a significant pollen donor effect from the variety “SIAL 70” which was associated with decreased palmitic acid levels.

## Introduction

The cacao tree (*Theobroma cacao* L.) provides the essential raw materials for the manufacture of chocolate, with the unique fatty acid (FA) profile of cocoa beans contributing to the desirable textural characteristics of chocolate and other confections. The center of *T. cacao* diversity is Western Amazonia (Motamayor et al., 2002; Cornejo et al., 2018) although the trees are now cultivated globally throughout the humid tropics. Small melon-like pods produce 25 to 50 seeds (“beans”), which are traditionally fermented in banana leaves, boxes, baskets, or bags. The microbial fermentation promotes the development of flavor and color to the beans, which are then dried in the sun. Manufacturers clean and roast the dried beans, during which decortication of the fibrous testa occurs and is removed through winnowing to extract the cotyledon. Husked and winnowed beans are cracked into smaller pieces (“nibs”) that are ground to produce cocoa liquor, which is roughly 45% to 55% cocoa butter, i.e., fat. Hydraulic presses or millstones extract the cocoa butter from the liquor, with the remaining cake becoming cocoa powder.

The FA composition of cocoa butter is roughly equal parts palmitic (C16:0), stearic (C18:0), and oleic (C18:1) acids (Lipp and Anklam, 1998; Lipp et al., 2001; Naik and Kumar, 2014). The saturated FAs, palmitic acid, and stearic acid have relatively high melting points of 62°C and 68°C, respectively, whereas unsaturated oleic acid melts at only 16°C (Berg, et al., 2002). This unique combination allows chocolate to be solid at room temperature, yet melt when it touches the palate (McHenry and Fritz, 1987). A minor fraction of the FA composition of cocoa butter contains the saturated arachidic acid (C20:0), the unsaturated palmitoleic acid (C16:0) and traces of the other FAs. The unique FA profile of cocoa butter also makes it desirable to the cosmetics and pharmaceutical industries, and it is one of the most expensive edible fats in the world (McHenry and Fritz, 1987).

**Figure 1:**
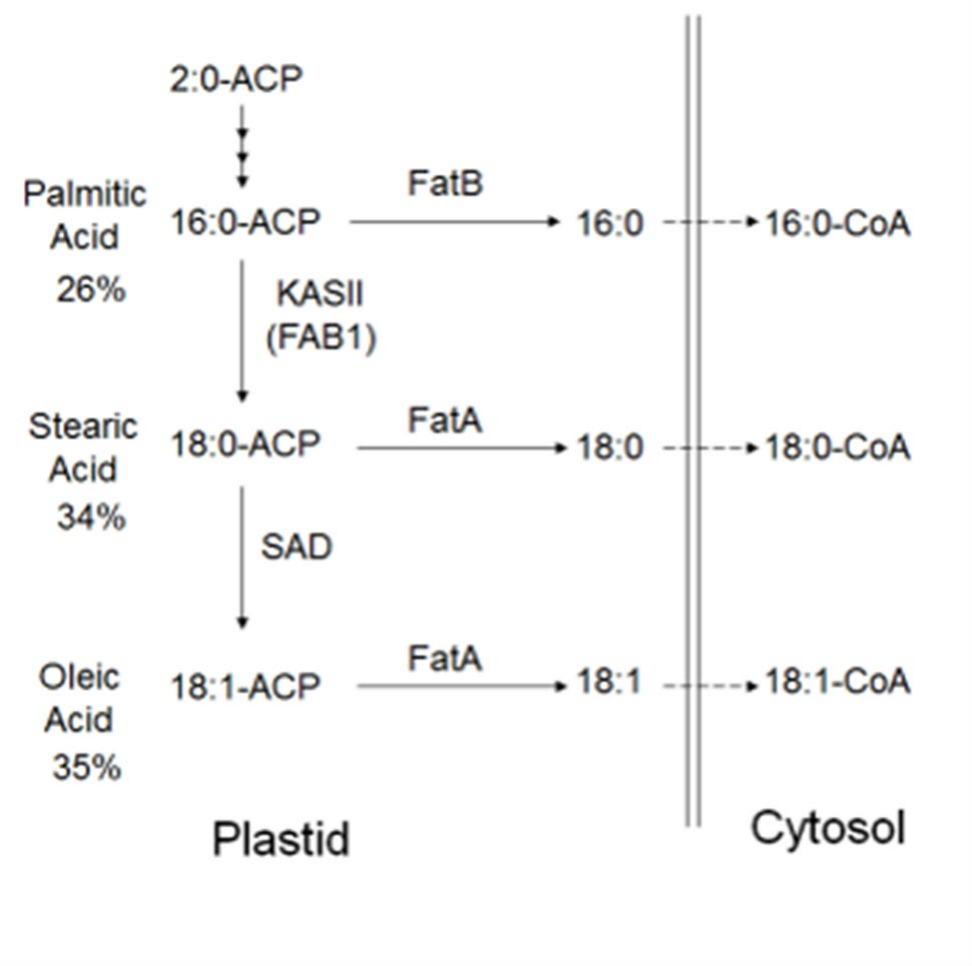
Predicted FA biosynthesis pathway in cacao with candidate genes. Cocoa bean fat is comprised primarily of palmitic acid, stearic acid, and oleic acid. FAs are synthesized in plastids, prior to transport to the cytosol, then the endoplasmic reticulum for modification and lipid assembly. Candidate genes in other plant species have been identified, and fatty acid-related genes exist (Argout et al., 2011)

In plants, the FA biosynthetic pathways are relatively well characterized (**Figure 1**). Long-chain FAs are formed by sequential condensation, reduction, dehydration, and reduction of two-carbon units by β-ketoacyl-acyl carrier protein (ACP) synthase (KAS family) enzymes (Ohlrogge and Browse, 1995) with the ACP. Each KAS enzyme has different specificities. KASIII condenses acetyl-CoA with malonyl-ACP to form 4:0-ACP (Li-Beisson et al., 2013). KASI elongates 4:0-ACP to 16:0-ACP. KASII elongates 16:0-ACP to 18:0-ACP (Pidkowich et al., 2007). The stromal Δ9 stearoyl-ACP desaturase (SAD) SAD2 desaturates 18:0-ACP to form 18:1-ACP. FATA and FATB are ACP thioesterases that terminate FA chain extension, releasing the FAs from ACPs, so that palmitic acid, stearic acid, and oleic acid can be exported from the plastid (Koo et al., 2004) for lipid assembly in the endoplasmatic reticulum. Biosynthesis from stearic (C18:0) to arachidic FA (C20:0) is achieved through elongases coded by FA elongation gene (FAE) whereby very long-chain FAs (VLCFA), such as C18:0, are synthesized with the addition of carrier proteins (Barker et al., 2007). In the cytosol, acyl chain elongation of the monounsaturated C18:1 to the polyunsaturated C18:2 is facilitated through FA desaturase 2 enzyme (FAD2) (Dar et al., 2017).

The effects of environmental conditions on FA composition have been consistently shown in many studies on various plant species (Yeilaghi et al., 2012; Bellaloui et al., 2015; Hemingway et al., 2015). Environmental conditions have also been shown to impact the ratio of saturated (palmitic and stearic acids) to unsaturated (oleic acid) FAs in cocoa beans, with the proportion of oleic and linoleic acids increasing with lower temperatures (Lehrian et al., 1980; McHenry and Fritz, 1987). South American origins, in particular Brazil, are associated with higher oleic, “soft” cocoa butter (Chaiseri and Dimick, 1989; Lipp and Anklam, 1998). “Soft” cocoa butter from cooler climates have a lower melting point (Lehrian et al., 1980), complicating chocolate manufacture, and are associated with lower prices for growers (McHenry and Fritz, 1987). 

Variation in cocoa bean FA composition and total fat levels has been observed between genotypes (Liendo et al., 1997; Pires et al., 1998; Vázquez-Ovando et al., 2015). The genetics of FA biosynthesis have been identified in Arabidopsis and are relatively conserved across plants (Lightner et al., 1994; Kachroo et al., 2007). The Belizian Criollo “B97-61/B2” genome had 84 orthologues to the 71 FA-associated Arabidopsis genes (Argout et al., 2011). The only published quantitative trait loci (QTL) mapping of total bean fat levels reported extreme transgressive segregation: Trinitario (“ICS 1”) and Contamana (“Scavina 6”) parents have similar fat levels (52% and 54%, respectively), but their F_2_ progeny averaged only 46% (Araújo et al., 2009). Although QTLs for FA composition and cocoa butter hardness were identified in the said population, both LOD scores were low (5 and 3, respectively), with large QTL regions occupying approximately one third of the linkage group.

Here, we report the use of a segregating mapping population from a cross between two heterozygous parents to identify QTLs explaining the variability of palmitic, stearic, and arachidic acid levels in cocoa beans. The female parent, “TSH 1188,” is a hybrid variety developed in Trinidad, and contains genetic material from “POUND 18,” “TSH 735,” and “TSA 641,” with this last one being the result of a cross between “SCA 6” and “IMC 67” (Turnbull and Hadley, 2019). The male parent, “CCN 51,” is likely the most commonly cultivated commercial hybrid clone in Latin America and is derived from crosses involving “IMC 67” and “ICS 95” (Boza et al., 2014). Our study shows a strong genetic effect on the FAs of cocoa beans, as indicated by high-log_10_ *p* scores found. We also report a significant role for environmental factors, with emphasis on temperature fluctuation and pollen donor effects in FA composition. 

## Materials and Methods

### Mapping Population

The creation of the MP01 mapping population, including the genotyping procedure and its genetic map, was described in Motamayor et al. (2013), Barreto et al. (2015), Royaert et al. (2016), and Stack et al. (2015). Briefly, this study involves 420 of the total 459 offspring from a cross between “TSH 1188” and “CCN 51.” The MP01 mapping population was planted at Mars Center for Cocoa Science in Barro Preto, Bahia, Brazil (14°42′45 N, 39°22′13 E) in the year 2000 in a 3 × 3 m grid in a hydromorphic and typical tropudalf (Itabuna modal) mixed soil type. Shade was provided using the traditional “cabruca” system, where the trees are grown amongst the Atlantic Forest’s native canopies. DNA was extracted from leaves using the protocol described by Motamayor et al. (2013) and ran on the cacao Illumina Infinium Cacao6kSNP array (Livingstone et al., 2015). JoinMap^®^4.1 (Van Ooijen, 2006) was used to create the genetic map, integrated genetic maps were obtained using the Maximum Likelihood (ML) mapping algorithm (Van Ooijen, 2011), map distances were calculated using the Haldane mapping function and the resulting maps were drawn using MapChart 2.1 (Voorrips, 2002).

### Bean Sampling

**Figure 2:**
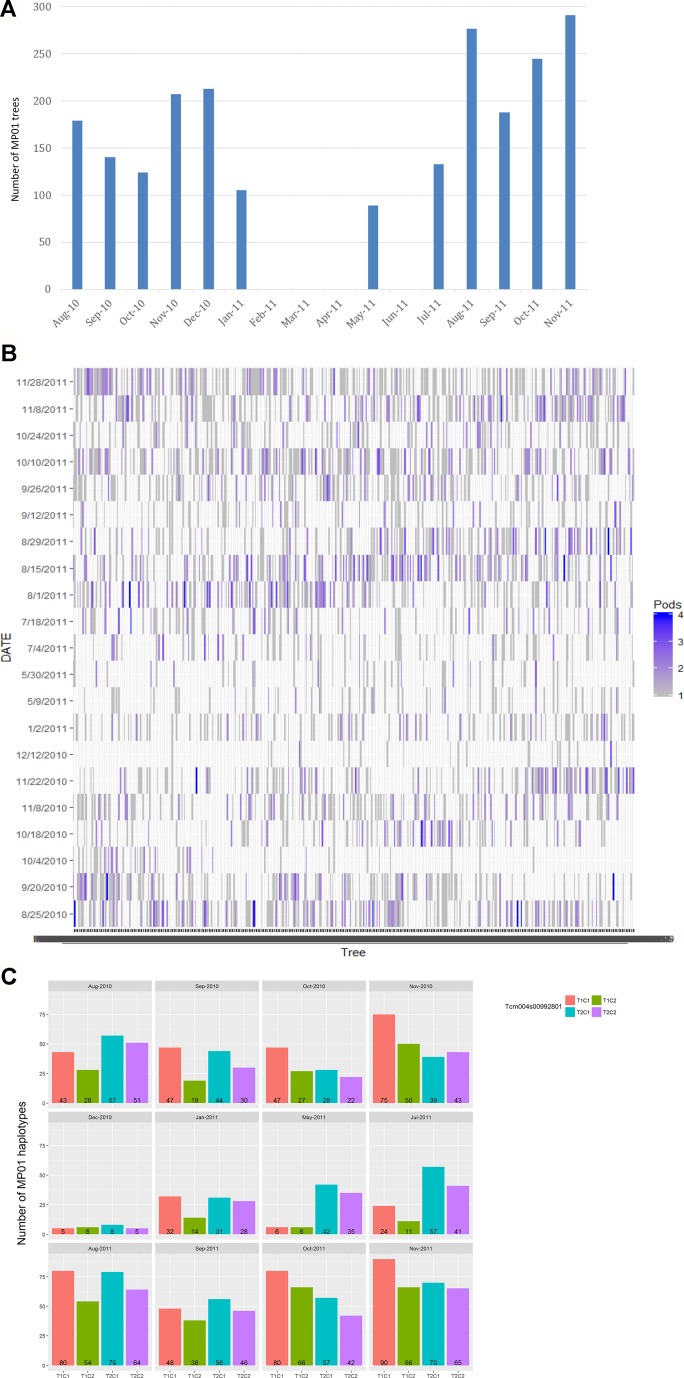
Harvest of MP01 progenies for FA analysis. **(A)** Distribution of number of MP01 progenies (y-axis) by month of harvest (x-axis). **(B)** Number of replicates (pods) per genotype (x-axis) harvested per harvest date (y-axis). **(C)** Skew of parental haplotype combinations (T1C1, T1C2, T2C1, and T2C2) based on harvest date, based on Tcm004s00992801 marker.

Two to four biological replicates were sampled for each genotype, based on mature pod availability across 12 harvest months with one to three harvest days per month from August 2010 to November 2011 (**Figure 2**). The parents, “TSH 1188” and “CCN 51” had one extra sampling date in July 2010. Biological replicates for individual trees came from multiple pods harvested from the same tree. Each sample contained about 13 beans (200g of wet bean) which were micro-fermented as described in Seguine et al. (2014). A total of 3,292 samples were analyzed for FA composition comprising of 420 total genotypes in the MP01 population.

### Liquor Processing

Pods from the MP01 population (“TSH 1188” × “CCN 51”) were harvested based on inspection of the pods and classified as ripe by experienced cacao harvesters. The harvested pods were free of any evidence of disease, particularly witches’ broom disease (WBD) and black pod (BP), two prevalent diseases in the Bahia region. Each pod was harvested separately, fermented and dried as described in the protocol of the micro-fermentation patent (Seguine et al., 2014). Following the fermentation and drying, the beans were stored for at least six weeks to stabilize the flavor of the beans. Storage was at ambient conditions in a screened area to avoid infestation.

After aging, the beans were roasted using a FD153 convection drying oven with multiple samples roasted on a split tray using separators. Beans were roasted one layer deep. Approximately 80 to 100 samples were roasted at a time (single pods, approximately 30–50 g of net dry beans per pod). Roasting was conducted at 120°C for 25 min timed from the time the oven temperature recovered to 118°C (range of recovery time, 5–7.5 min). Following the roasting, the entire tray was put out in the lab on risers that held it approximately 3 inches on the top of a stainless steel lab table with air flow from the top. Beans were cooled to room temperature for approximately 30 min, then placed individually into small polyethylene bags, and held not more than 24 h before subsequent processing.

For processing, the beans were placed in a small plastic bag and crushed with a rolling pin to separate the shells from the beans and to break them down into smaller pieces. Individual samples were winnowed with a CAPCO Engineering Cocoa Winnower and the winnowed nibs were inspected for shells and residual shells were removed.

Milling was then conducted in a RETSCH-RM200 motorized mortar and pestle equipped with porcelain mortar and pestle. The mill was run for a total of 30 min with scraping at the 5- to 10-minute mark to ensure complete grinding of the entire nibs. Following the grinding, particle size was measured with a micrometer to obtain fineness within the range of 18 to 25 microns. Mass was transferred to high-density polypropylene centrifuge tubes and capped. Liquor samples were put into tubes, varying between 15 and 30 g. Tubes were stored for one week at ambient conditions and then moved to a −80ºC freezer until analysis.

A total of 3,292 cocoa liquor samples were analyzed for FA composition comprising of 420 total genotypes in the MP01 population. The preparation of liquor samples followed all of the defined parameters recommended by the cocoa ECA/CAOBISCO/FCC quality guide (CABISCO/ECA/FCC, 2015) which defines in a general sense all of the protocols used.

### Bean Fat Analysis

Approximately 25 mg of cocoa liquor samples were weighed in 13 x 100 mm screw cap glass tubes. Furthermore, 2.5 ml of 2.5% (v/v) sulfuric acid in methanol and 60 mg of the internal standard triheptadecanoin (17:0-triacylglycerol; NuChek Prep) from a 10 mg/ml stock solution dissolved in toluene were added. Samples were capped under nitrogen and mixed thoroughly prior to heating at 95°C for 1 h. Following cooling, 3 ml of heptane and 3 ml of deionized water were added to each tube. These were mixed thoroughly and centrifuged at 1000*g* for 2 min in a Q222 RM table top centrifuge (Quimis, Diadema, Sao Paulo, Brazil). The recovered heptane layer containing FA methyl esters was transferred to autosampler vials and analyzed by gas chromatography using an Agilent 7890 gas chromatograph (GC) with flame ionization detection (FID). Sample components were resolved using a HP-INNOWax column (30 m length × 0.25 mm inner diameter × 0.25 µm film thickness; Agilent) with oven, detector, and injector temperatures as described in Cahoon et al. (2006). Briefly, the GC inlet and FID temperatures were set at 250°C and 260°C, respectively. The oven was operated at a temperature program of 185°C (1 min hold) to 230°C (3 min hold) over a linear gradient of 7°C/min. The carrier gas was H_2_ at an inlet flow rate of 30 ml/min. FA compositions of samples were obtained from these analyses. Fat content was obtained by quantifying FA methyl esters in samples by measurement of FID response relative to that of methyl heptadecanoate from the internal standard (Christie, 1982; Badings and De Jong, 1983; Christie, 1989). 

### Identification of QTLs

QTL mapping analysis for the FAs was performed on the MP01 linkage map with Genstat v.18 (VSN International, 2015). Each trait was checked for normality and mapped with the interval mapping (IM) procedure. After the initial IM, the markers with highest *P* values obtained from Wald tests in the -log_10_ scale (-log_10_ *p*) were used as cofactors in the subsequent QTL run as part of the composite IM (CIM) procedure. A second analysis with the REML algorithm for linear mixed models was used to separate the additive parental effects and dominance effect (interaction). For each marker, the associated probability (*p* value) from the Wald test statistic was used to test significance (Lynch and Walsh, 1998). The threshold for significance used was the corrected-Bonferroni option in Genstat (Li and Ji, 2005) with 0.05 significance level. For these analyses the threshold for maternal, paternal and interaction effects are 3.688, 3.701, and 3.826, respectively.

QTL analyses were performed on two instances/partitions of the phenotypic data: (i) best linear unbiased predictors (BLUPs) obtained from pooled data with the 12 sampling dates from August 2010 to November 2011, and (ii) BLUPs for the effects of genotypes by harvest season. The two harvest seasons analyzed spanned the periods August 2010 to January 2011 for the first harvest season, and May 2011 to November 2011 for the second harvest season. Both harvest seasons have 6 months of pod collection, with data for 361 genotypes on the first year, 404 genotypes in the second year, and 420 genotypes for the combined periods. Weather data from the MCCS research station were used to complement the data for both harvest seasons. These data include daily average, minimum and maximum temperatures in °Celsius and rainfall in millimeters. Summary statistics for the climate data are presented in **Supplementary Table 1**. The average FA and fat content were calculated by date of harvest, generating twelve data points. The FA means were then compared to the corresponding average temperatures of the months prior, where one lag (lag1) indicates comparison between the FA means of trees harvested in a given month to the average temperatures 1 month prior.

### Haplotype Phasing and Effect

The parental haplotypes were identified for each of the MP01 progeny for both the peak markers and the markers at the regions surrounding the QTLs associated with FAs, for the two partitions of the data previously described. The genotypes of the progeny were phased with iXora (Utro et al., 2013) and with JoinMap (Van Ooijen, 2011). Favorable alleles/haplotypes were identified by taking the average percentage of FAs of the individuals containing the parental haplotype combinations separately for each FA peak marker. To test the significance of the haplotype–phenotype associations, a regression was applied to the peak markers, and the Tukey post hoc test for multiple comparisons was used to identify the difference in the effect of the parental haplotype combinations (T1C1, T1C2, T2C1, and T2C2), where “T” and “C” represent the parental haplotypes from “TSH 1188” and “CCN 51,” respectively.

### Phylogenetic Analysis of Palmitic Acid Allele

A diversity panel consisting of 200 accessions were re-sequenced at high coverage (Cornejo et al., 2018) and filtered to match markers in common with the 6K SNP chip used for MP01. The filtered genotypes were then phased with the program Beagle v4 (Browning and Browning, 2007), with 10 burn-in iterations, 5 iterations for burn-in phase, sliding window of 1000 with 500 overlaps, and 16 threads for haplotype sampling. Twenty-two markers were used within the QTL region encompassing one-log_10_ *p* away from the peak marker for palmitic acid in chromosome 4. The distance matrix for phylogeny estimation was created with the Maximum Composite Likelihood algorithm in MEGA v.7.0 with 1000 bootstraps (Kumar et al., 2016) for the 400 haplotypes. The resulting neighbor-joining (NJ) was collapsed to reflect support values of 70% or higher from the bootstraps.

#### Structure Analysis of 200 Genomes Diversity Panel

The 200 accessions were assigned to the 10 genetic cacao groups (Motamayor et al., 2008; Cornejo et al., 2018) by proportion of membership, using a supervised admixture analysis with Admixture software (Alexander et al., 2009). The reference set for the cacao groups consisted of genotypes with 85% or more membership to a specific genetic group.

### Statistical Analysis

Summary statistics of the FA composition for the parents, “TSH 1188” and “CCN 51” with *p* value from Student’s *T* distribution testing the null hypothesis of equal means. The significance of the genotypic variation was determined with a multivariate analysis of variance (MANOVA) for tests of main effects, including genotype, pollination type, and harvest season. Univariate ANOVAs were generated from the multivariate linear model, corrected for simultaneous inference with the Holm *p* value adjustment.

In addition, a random effects model was used to estimate the variance components of the tested traits:

$$\begin{array}{l} Model:\;y_{ijk}=\mu +G_{i}+\alpha_{j}+{\left(G\alpha \right)}_{ij}+\varepsilon_{ijk}\\ \;G_{i}∼N(0,\;\sigma_{G}^{2}),\;\alpha_{j}∼N(0,\;\sigma_{y}^{2}),\;{\left(G\alpha \right)}_{ij}∼N(0,\;\sigma_{Gy}^{2})\\ \;and\;\varepsilon_{ijk}∼N(0,\;\sigma_{\varepsilon }^{2}) \end{array}$$

where ***y*** ***_ijk_*** is the individual FA observation of the *k* ^th^ sample (*k* = 1,…,9) of the *i* ^th^ F1 genotype in the *j* ^th^ harvest season, ***µ*** is the grand mean, **ε** ***_ijk_*** is the residual variance, ***G*** ***_i_*** is the random effect of the **i** ^th^ genotype (*i* = 1,…, 413) , **α** ***_j_*** is the random effect of *j*th harvest season (*j* = 1,2), and (**Gα**)**_ij_** is the genotype-by-harvest season interaction for the *i*th genotype in the *j*th year. The estimates of the variance components were obtained with the above model and ran with the program ASREML-R (Butler et al., 2009) with the restricted maximum likelihood method. The standard errors for the ratios of the variance components were obtained by approximation with the Delta method and the BLUPs were used in the QTL analyses.

Broad-sense heritability (***H*** **^2^**) and the total phenotypic variance (${\hat{\sigma }}_{p}^{2}$) are expressed as:

$${\hat{\sigma }}_{p}^{2}={\hat{\sigma }}_{G}^{2}+{\hat{\sigma }}_{y}^{2}+{\hat{\sigma }}_{G*y}^{2}+{\hat{\sigma }}_{\varepsilon }^{2}$$

$$H^{2}={\hat{\sigma }}_{G}^{2}/{\hat{\sigma }}_{p}^{2}$$

The proportions of each variance component estimate are expressed as ${\hat{\sigma }}_{y}^{2},\;{\hat{\sigma }}_{G*y}^{2}$ and ${\hat{\sigma }}_{3}^{2}$ ${\hat{\sigma }}_{\varepsilon }^{2}$ relative to the total phenotypic variance ${\hat{\sigma }}_{p}^{2}$.

## Results

### Variation in Cocoa Butter Fat Levels and FA Composition

Extensive variation was found in the progeny of the MP01 population: The effect of genotype was highly significant in the MANOVA (*p* < 0.0001) with Wilk’s λ = 0.01 and in the univariate case and was found to be significant in all the FAs and percentage of fat content (**Table 1**). The parents, “TSH 1188” and “CCN 51,” show nearly identical FA composition in our data set, but with “TSH 1188” having slightly higher average fat content levels (**Supplementary Table 2**).

**Table 1:** Multivariate analysis of variance (MANOVA) and univariate ANOVA for FA values (C16:0 Palmitic, C18:0 Stearic, C18:1 Oleic, C18:2 Linoleic, C20:0 Arachidic) represent proportion of total FA for the specific FAs.

Trait	Source of variation	Wilk’s λ	*df*	Num *df*	den *df*	*p*
All	Genotype	0.01	419	2514	5331	<0.0001
All	Pollination type	0.51	2	12	1774	<0.0001
All	Harvest season	0.64	1	6	887	<0.0001
		**SS**	***df***	**MS**	***F***	**Holm ** ***p***
C16:0	Genotype	1856.13	419	4.43	4.36	<0.0001
C16:0	Pollination	547.69	2	273.85	1.29	0.00324
C16:0	Harvest season	4.23	1	4.23	0.01	0.9999
C16:0	Residuals	905.73	892	1.02		
C18:0	Genotype	1955.74	419	4.67	856.75	<0.0001
C18:0	Pollination	459.60	2	229.80	201.34	<0.0001
C18:0	Harvest season	179.26	1	179.26	78.53	<0.0001
C18:0	Residuals	1018.11	892	1.14		
C18:1	Genotype	779.32	419	1.86	964.08	<0.0001
C18:1	Pollination	52.12	2	26.06	64.48	<0.0001
C18:1	Harvest season	165.45	1	165.45	204.68	<0.0001
C18:1	Residuals	721.05	892	0.81		
C18:2	Genotype	274.54	419	0.66	3.51	<0.0001
C18:2	Pollination	3.02	2	1.51	0.04	0.9999
C18:2	Harvest season	5.95	1	5.95	0.08	0.9999
C18:2	Residuals	166.52	892	0.19		
C20:0	Genotype	5.23	419	0.01	591.82	<0.0001
C20:0	Pollination	0.12	2	0.06	13.44	<0.0001
C20:0	Harvest season	0.74	1	0.74	83.7	<0.0001
C20:0	Residuals	3.94	892	0.00		
% Fat	Genotype	2917.24	419	6.96	769.31	<0.0001
% Fat	Pollination	4.72	2	2.36	1.24	0.5298
% Fat	Harvest season	429.78	1	429.78	113.34	<0.0001
% Fat	Residuals	3382.49	892	3.79		

Percent (%) fat is the percent of total fat in the bean. Tests for effects of genotypic variation (genotype), pollination (open pollinated, self-pollinated or pollen donor “SIAL 70”), and harvest season (two seasons), Wilk’s λ (multivariate test statistic), degrees of freedom (df), numerator (num df), and denominator degrees of freedom (den df), type III sum of squares, mean square (MS), F statistic (F value), and Holm’s adjusted P value (Holm p) for univariate ANOVA.

**Figure 3:**
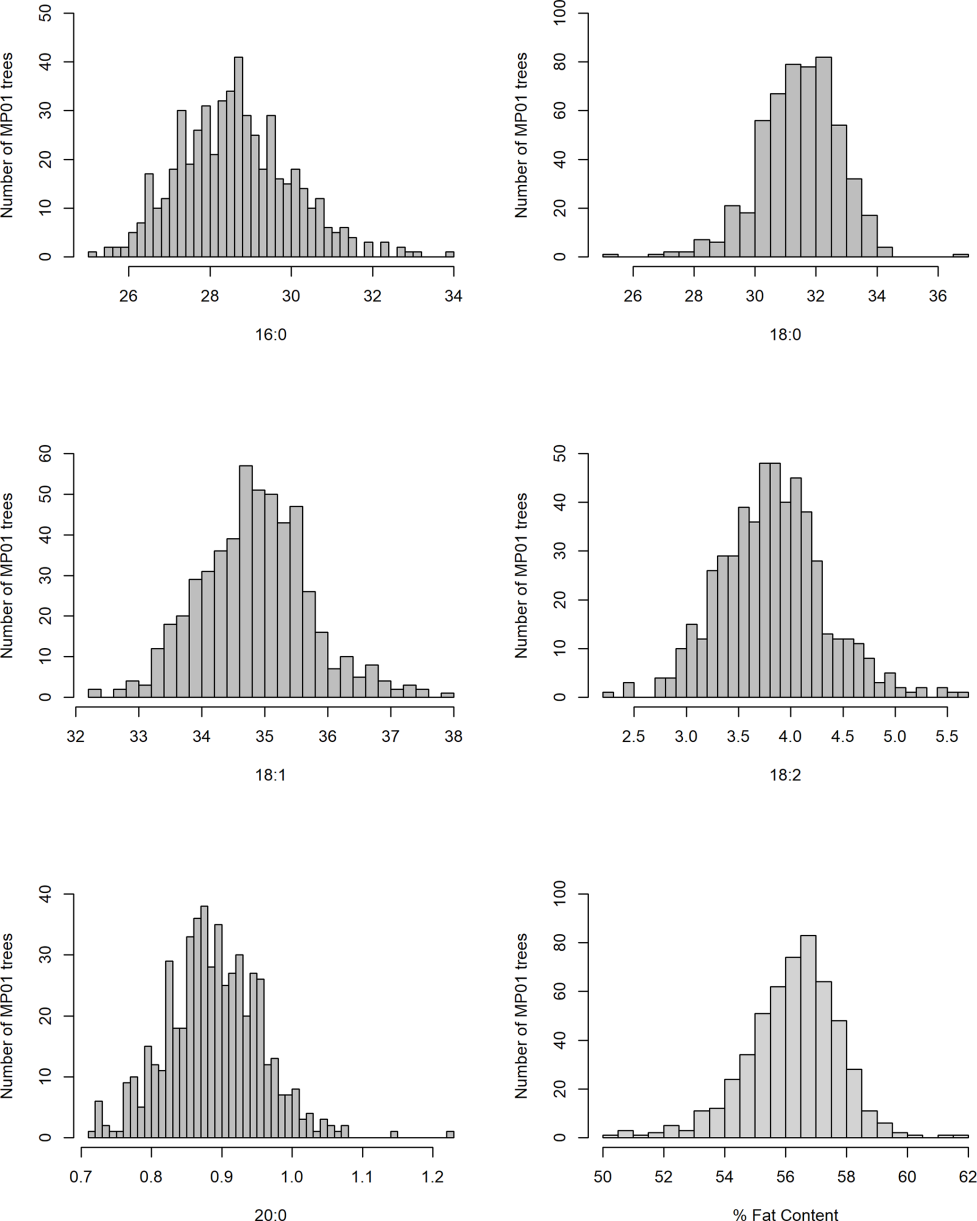
Distribution of FA content for MP01 progenies for both periods.

In our study, total fat content in the progeny ranged from 32.2% to 70.7% and from 50.2% to 62.4% when averaged per genotype (**Table 2** and **Figure 3**). The mean fat content for the mapping population was 56%, falling between the means of the two parents, “TSH 1188” and “CCN 51” (54.6% and 57.3%). The level of palmitic, stearic, and oleic acids represents a total of 94.9% of FAs (**Table 2**). While the observed levels for the two major saturated FAs, palmitic and stearic acids, were 25.6% and 25.1%, respectively, for the monounsaturated oleic acid, the lowest level observed was 32.6%. There was also a much smaller range of variation for oleic acid, with only 5.2 percentage points separating the maximum and minimum genotypes. Ranges for palmitic and stearic acid were 8.2% and 11.3%, respectively.

**Table 2:** Variation in MP01 beans.

Statistic	C16:0	C18:0	C18:1	C18:2	C20:0	% Fat Content
# obs	420	420	420	420	420	420
Min	25.6	25.1	32.6	2.3	0.7	50.2
Max	33.8	36.3	37.8	5.6	1.2	62.4
Range	8.2	11.3	5.2	3.4	0.5	12.3
Median	28.7	31.4	34.9	3.9	0.9	56.2
Mean	28.7	31.3	34.9	3.9	0.9	56
Std.dev	1.4	1.4	0.9	0.5	0.1	1.6
**Quantile**						
0%	25.6	25.1	32.6	2.3	0.7	50.2
25%	27.7	30.4	34.3	3.5	0.8	55.1
50%	28.7	31.4	34.9	3.9	0.9	56.2
75%	29.5	32.2	35.4	4.1	0.9	56.9
100%	33.8	36.3	37.8	5.6	1.2	62.4

Values represent the summary statistics per genotype. FA values (C16:0 Palmitic, C18:0 Stearic, C18:1 Oleic, C18:2 Linoleic, C20:0 Arachidic) represent proportion of total FA for the specific FAs. Percent (%) fat content is the percent of total fat in the bean.

### Correlation Between Traits

The main significant correlations were negative correlations between palmitic acid and stearic acid (−0.74), and between palmitic acid and oleic acid (−0.37) (**Table 3**). A negative correlation between palmitic acid and arachidic acid (−0.37) was also identified, but of less biological interest, given that arachidic acid represents less than 1% of FAs in cocoa beans (**Table 2**). Fat content (as a percentage of the bean) was positively correlated with stearic acid levels (0.25) and negatively correlated with palmitic (−0.12) and oleic acid levels (−0.11). Directionality of the FA traits and total fat content are displayed in the PCA plot (**Supplementary Figure 1**), capturing 57.9% of the total variation in the data.

**Table 3:** Correlations between fat traits (least squares means).

	C16:0	C18:0	C18:1	C18:2	C20:0	% Fat Content
**C16:0**						
**C18:0**	−0.74***					
**C18:1**	−0.37***	−0.24***				
**C18:2**	−0.05	−0.33***	0.01			
**C20:0**	−0.37***	0.39***	−0.16**	0.13*		
**% Fat Content**	−0.12*	0.25***	−0.11*	−0.20***	0.12*	

Pearson’s correlation coefficient matrix of genotype means for FA levels and total fat content of the bean. The darker gradients represent stronger correlation and the lighter colors represent weaker correlations. Strength of test (H0: pair-wise correlation is 0) is represented by asterisks p < 0.0001 (***), p < 0.001 (**), p < 0.05 (*). p Values used the “Holm” adjustment for multiple comparisons.

**Figure 4:**
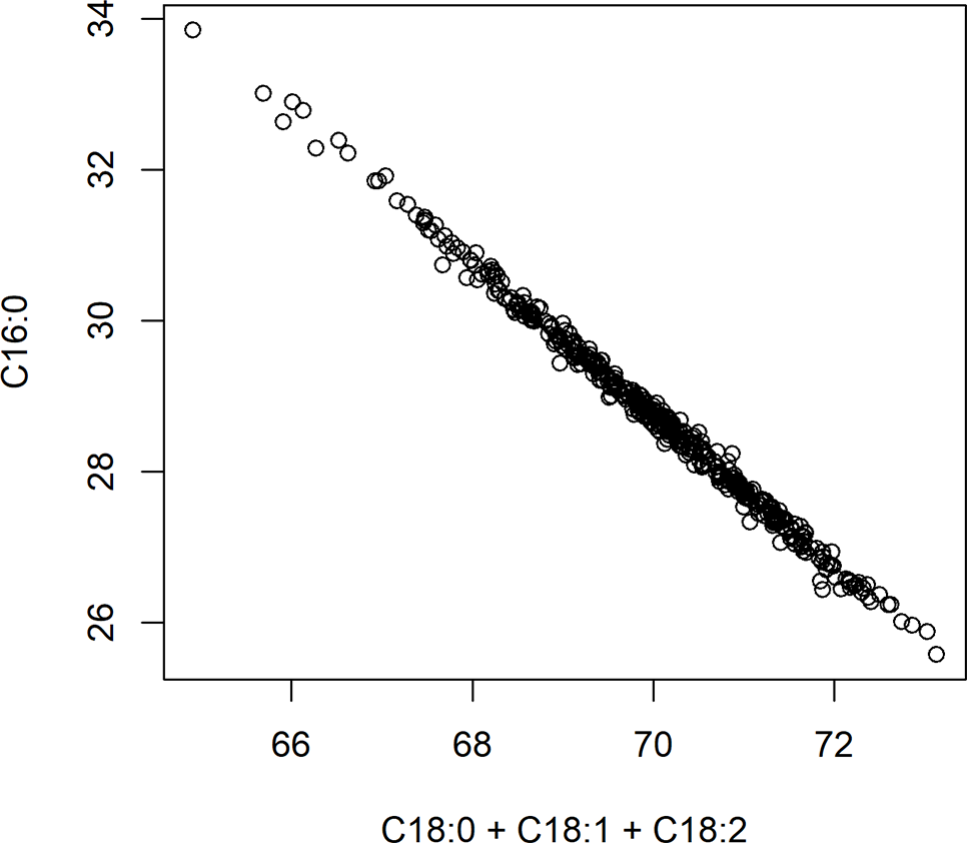
Negative correlation between palmitic acid (C16:0) and the sum of stearic acid (C18:0), oleic acid (C18:1), and linoleic acid (C18:2). Axes represent percent of either palmitic acid relative to total fat, or the sum of stearic, oleic, and linoleic acids relative to total fat.

A significant negative correlation was measured between the level of palmitic acid and the downstream FAs stearic (−0.74), oleic (−0.37), and linoleic acids (−0.05) (**Table 3**). In **Figure 4**, a graphical presentation of the negative correlation between the levels of palmitic acid and the sum of the downstream FAs is shown. This supports the predicted cacao FA biosynthesis pathway, where palmitic acid is consumed by a KASII/FAB1 enzyme to create stearic acid and then can be converted into oleic acid by SAD/FAB2 (**Figure 1**).

### Sampling of MP01 and Identification of QTLs

The collection of samples depended on pod availability, which varied between sampling time and genotypes. For this reason, the pooled data from all months were used for the QTL analyses, and the 12 months were partitioned into two harvest seasons (**Figure 2**) to determine a variance component for environment in terms of seasonal effect.

**Figure 5:**
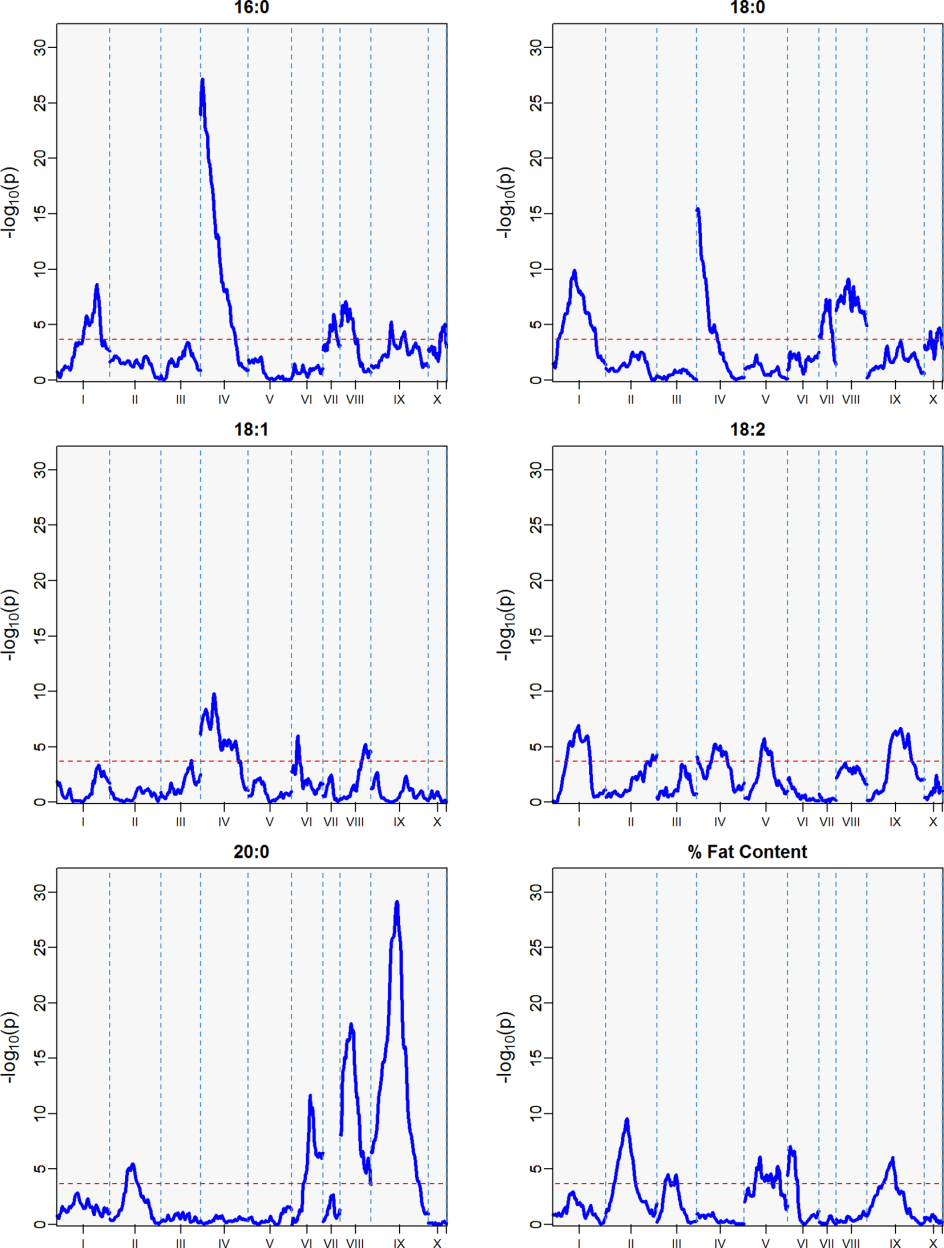
QTLs from best linear unbiased predictors (BLUPs) calculated using the 12 sampling dates spanning August 2010 to November 2011. The -log_10_ *p* is the statistical magnitude of the significance of the peak maker associated to the trait. The coordinates are based on the “Matina1-6” genome V1.1. The threshold of significance (red dash) is a multiple comparison adjustment at 3.688.

QTLs with a -log_10_ *p* score greater than the threshold of 3.688 were identified. The IM and CIM mapping procedures were similar in outcome for the position of the peak markers in the traits evaluated, thus we report the IM results from the haplotype phase from the QTL regions encompassing one -log_10_ *p* away from the peak markers (**Table 4**, **Figure 5**).

**Table 4:** QTL results from the IM procedure for combined FA BLUP values for the combined harvest seasons.

Trait	LG	-log_10_ *p*	% var. expl	Range (Mbps)	Peak marker	Start	Stop	T1C1 Tukey	T1C2 Tukey	T2C1 Tukey	T2C2 Tukey	T1C1	T1C2	T2C1	T2C2	T1	T2	C1	C2
C16:0	1	8.8	8.9	1.6	32,918,760	32,918,760	34,560,615	a	a	b	c	28.3	28.3	28.9	29.4	28.3	29.1	28.6	28.9
C16:0	4	27.6	23.5	0.4	992,801	651,315	1,006,787	a	a	b	b	28.1	28.1	29.3	29.6	28.1	29.4	28.7	28.8
C16:0	7	6.0	6.2	5.7	7,581,095	4,489,509	10,151,992	ab	b	a	a	28.8	29.3	28.6	28.3	29.0	28.4	28.7	28.8
C16:0	8	7.1	7.3	0.3	1,169,653	869,653	1,469,653	ab	c	a	bc	28.7	29.2	28.2	28.9	28.9	28.5	28.4	29.1
C16:0	9	5.8	5.9	0.4	6,156,866	6,064,842	6,463,616	a	a	a	b	28.5	28.5	28.6	29.3	28.5	29.0	28.6	28.9
C16:0	10	5.2	5.4	1.8	22,559,001	22,428,849	24,223,155	ab	a	bc	c	28.6	28.3	28.9	29.2	28.4	29.0	28.7	28.7
C18:0	1	10.0	10.0	2.9	10,168,848	8,353,112	11,276,895	b	b	b	a	31.9	31.6	31.5	30.6	31.7	31.1	31.7	31.0
C18:0	4	16.3	15.4	0.3	311,794	11,794	611,794	b	b	a	a	31.8	32.0	30.9	30.7	31.9	30.8	31.4	31.4
C18:0	7	7.3	7.4	3.3	4,402,504	4,369,124	7,678,456	ab	a	bc	c	31.1	30.9	31.6	32.0	31.0	31.8	31.3	31.4
C18:0	8	9.3	9.4	0.4	2,743,235	2,743,235	3,163,772	a	a	b	a	31.3	31.0	32.0	31.3	31.1	31.7	31.7	31.1
C18:0	9	3.8	3.9	0.3	33,349,545	33,049,545	33,649,545	b	a	b	a	31.6	31.1	31.6	31.0	31.4	31.4	31.6	31.1
C18:0	10	4.7	4.9	21.1	21,842,572	3,163,433	24,223,155	b	b	a	a	31.8	31.9	31.1	31.0	31.8	31.1	31.5	31.5
C18:1	1	4.0	4.1	0.3	34,641,359	34,341,359	34,941,359	b	ab	a	a	35.0	34.8	34.6	34.7	34.9	34.6	34.8	34.7
C18:1	3	4.5	4.7	0.3	30,259,960	30,259,960	30,606,185	b	b	ab	a	35.0	34.9	34.8	34.5	34.9	34.6	34.9	34.7
C18:1	4	10.0	10.0	1.4	16,507,100	16,210,309	17,636,309	b	b	a	a	35.0	35.0	34.6	34.4	35.0	34.5	34.8	34.7
C18:1	6	6.2	6.4	3.0	13,900,060	13,545,822	16,515,632	bc	a	c	ab	34.9	34.5	35.1	34.6	34.7	34.8	35.0	34.6
C18:1	8	5.3	5.5	6.0	15,120,872	9,610,444	15,631,835	b	b	a	ab	34.9	35.0	34.5	34.7	34.9	34.6	34.7	34.9
C18:2	1	7.6	7.7	1.0	12,340,943	12,200,052	13,162,985	a	bc	b	c	3.6	3.9	3.8	4.0	3.8	3.9	3.7	4.0
C18:2	2	5.5	5.7	0.3	41,100,466	40,846,923	41,141,331	bc	c	ab	a	3.9	4.0	3.8	3.7	3.9	3.7	3.8	3.8
C18:2	4	5.6	5.7	3.6	21,656,111	21,656,111	25,267,317	a	a	b	a	3.8	3.7	4.0	3.8	3.8	3.9	3.9	3.7
C18:2	5	5.9	6.1	8.2	28,110,818	24,696,466	32,855,680	a	b	a	a	3.8	4.1	3.7	3.8	3.9	3.8	3.8	4.0
C18:2	9	7.2	7.4	0.3	37,832,439	37,532,439	38,132,439	b	a	a	a	4.0	3.8	3.8	3.7	3.9	3.7	3.9	3.7
C20:0	2	5.7	5.9	3.5	7,662,068	5,894,024	9,348,946	c	ab	bc	a	0.9	0.9	0.9	0.9	0.9	0.9	0.9	0.9
C20:0	4	18.8	17.3	6.6	19,637,361	19,637,361	26,233,319	a	a	a	a	0.9	0.9	0.9	0.9	0.9	0.9	0.9	0.9
C20:0	6	11.7	11.5	0.2	23,105,784	23,105,784	23,272,507	c	ab	b	a	0.9	0.9	0.9	0.9	0.9	0.9	0.9	0.9
C20:0	8	18.4	17.0	1.2	3,274,675	2,113,570	3,274,675	b	a	b	a	0.9	0.9	0.9	0.9	0.9	0.9	0.9	0.9
C20:0	9	31.8	26.1	0.5	8,949,637	8,689,672	9,216,013	c	b	b	a	0.9	0.9	0.9	0.8	0.9	0.9	0.9	0.9
% Fat Content	2	9.8	9.8	1.2	7,032,556	6,637,416	7,831,310	c	bc	b	a	56.7	56.3	56.1	55.5	56.5	55.8	56.5	55.9
% Fat Content	3	4.6	4.8	12.7	24,615,758	12,502,277	25,250,690	ab	c	a	bc	55.7	56.6	55.6	56.3	56.2	56.0	55.7	56.5
% Fat Content	4	4.1	4.2	6.6	19,637,361	19,637,361	26,233,319	a	a	a	a	56.1	56.6	56.3	56.0	56.3	56.1	56.2	56.3
% Fat Content	5	6.2	6.4	9.8	8,884,977	8,884,977	18,719,781	b	a	b	a	56.5	55.8	56.6	55.9	56.2	56.2	56.6	55.8
% Fat Content	6	7.2	7.4	1.4	3,766,082	3,512,802	4,938,058	ab	c	a	bc	56.0	56.8	55.7	56.4	56.3	56.1	55.8	56.6
% Fat Content	9	6.6	6.7	0.6	9,216,013	8,689,672	9,263,083	b	a	ab	a	56.7	55.9	56.3	55.8	56.3	56.1	56.5	55.8

The -log_10_ p is the statistical magnitude of the significance of the peak maker associated to the trait. Range is the size in mega base pairs of the QTL region one-log_10_ p value away from the peak marker. Start and Stop indicate the locations for the range. Tukey’s multiple comparisons of haplotype differences are reported for each haplotype, where different letters refer to statistically different means at α = 0.05. The means for the genotypes belonging to the haplotype groups T1C1, T1C2, T2C1, and T2C2 are reported in addition to single haplotype means (T1, T2, C1, and C2). The coordinates are based on the “Matina1-6” genome.

The strength of the combined data set allowed us to identify two QTLs with -log_10_ *p* > 27 (**Table 4**). A QTL in LG 4 with a -log_10_ *p* of 27.6 explained 23.5% of variation in palmitic acid (C16:0), and a QTL on LG 9 with a -log_10_ *p* of 31.8 explained 26.1% of arachidic acid (C20:0) variation. Because they explain over 15% of the variation for the trait, we refer to these QTLs as “major.” Minor QTLs were identified for stearic acid (C18:0) (LGs 1, 4, 7, 8, 9, and 10), oleic acid (C18:1) (LGs 1,3,4,6 and 8), linoleic acid (C18:2) (LGs 1, 2, 4, 5, and 9), arachidic acid (C20:0) (LGs 1, 2, 4, 6, and 8), and % fat content (LGs 2, 3, 4, 5, 6, and 9).

The major QTL for palmitic acid (-log_10_ *p* > 15) was located in the gene model region, Tc04_g005590, corresponding to the SAD5 gene and minor QTLs (-log_10_ *p* ∼7) were located in the gene model regions Tc04_g01710, Tc04_g01720, and Tc04_g01740, corresponding to SAD1, SAD2, and SAD3 genes, respectively (Zhang et al., 2015). Significant markers for stearic acid were found only in the Tc04_g005590 region (-log_10_ *p* ∼9), which encodes for chloroplast transient peptides (Kachroo et al., 2004; Zhang et al., 2015). Oleic and linoleic acids had minor QTLs in all previously mentioned gene models, and a major QTL in LG 4 for arachidic acid which was found in the regions corresponding to gene models Tc04_g01710, Tc04_g01720, and Tc04_g01740. The gene model regions refer to the Criollo v1 assembly (Argout et al., 2011) and the Criollo v2 assembly annotation and positions (Argout et al., 2017) are also listed in **Supplementary Table 3**. In addition, the corresponding mapping of *SAD* gene models in the identified FA QTLs near the *FAB2/SAD* candidate genes are presented in **Supplementary Table 4**.

### Identification of QTLs by Harvest Season

**Figure 6:**
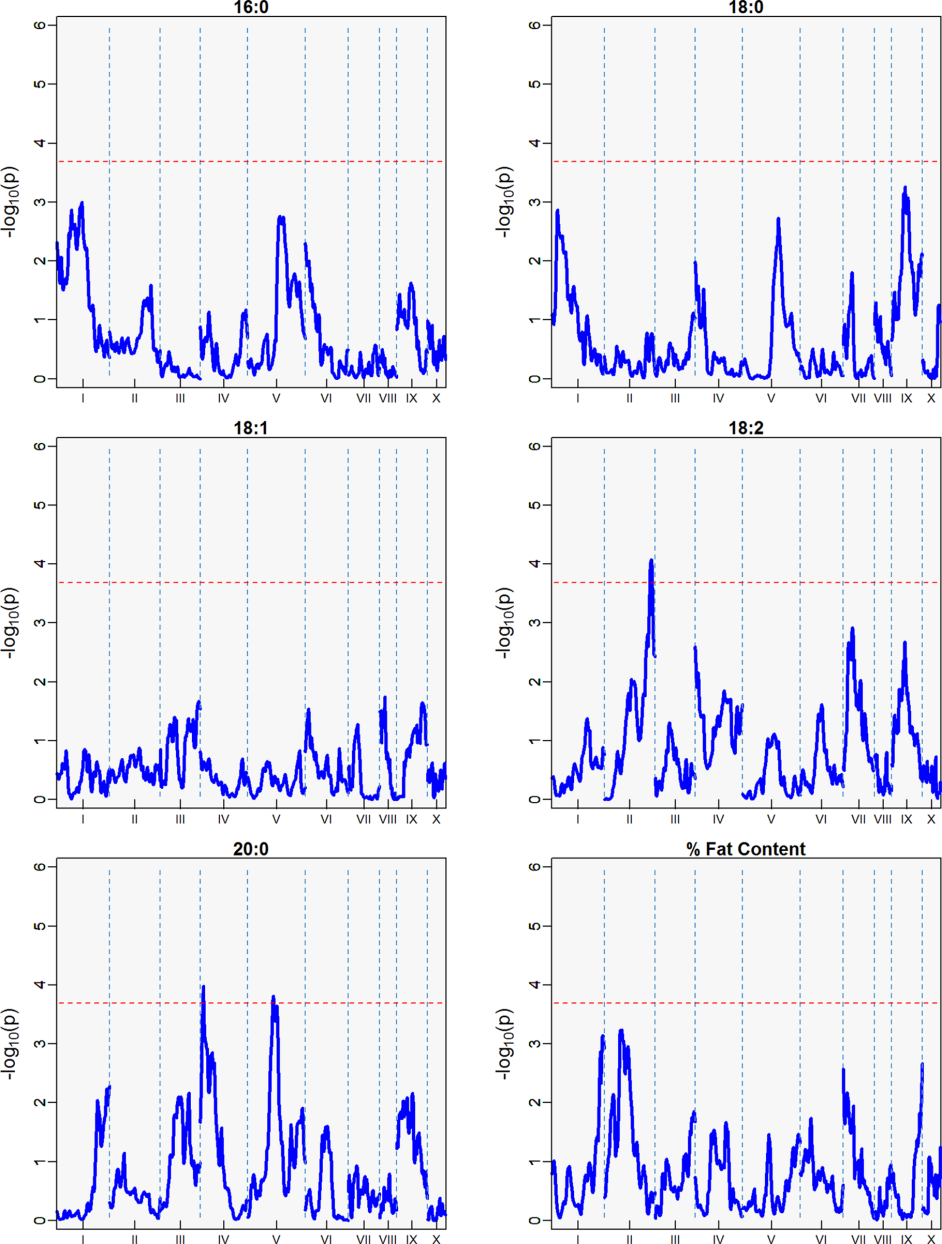
QTLs from the best linear unbiased predictors (BLUPs) from the first harvest season (6 sampling dates) spanning August 2010 to January 2011. The -log_10_ *p* is the statistical magnitude of the significance of the peak maker associated to the trait. The coordinates are based on the “Matina1-6” genome V1.1. The threshold of significance (red dash) is a multiple comparison adjustment at 3.688.

**Figure 7:**
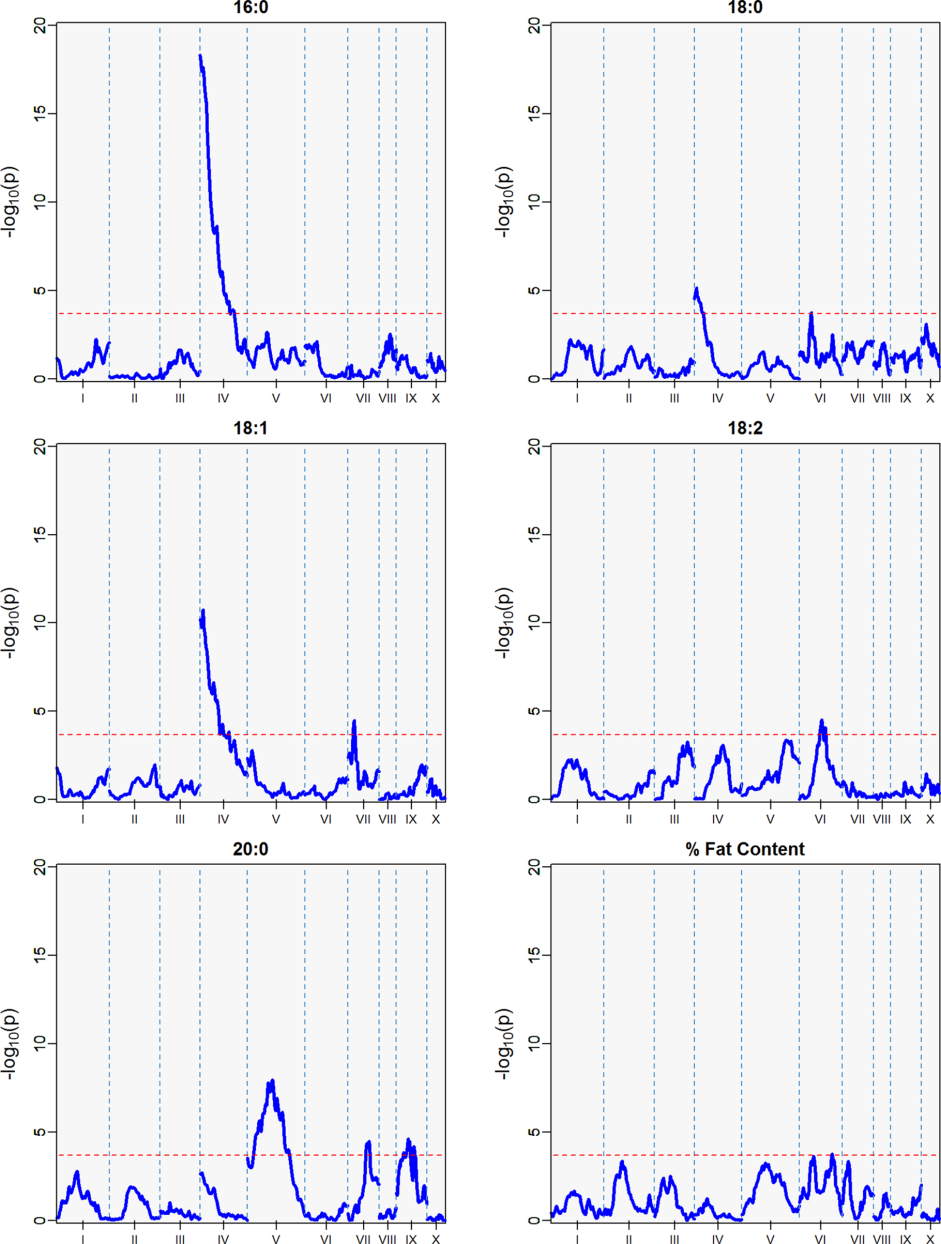
QTLs from the best linear unbiased predictors (BLUPs) from the second harvest season (6 sampling dates) spanning May 2011 to November 2011. The -log_10_ *p* is the statistical magnitude of the significance of the peak maker associated to the FA. The coordinates are based on the “Matina1-6” genome V1.1. The threshold of significance (red dash) is a multiple comparison adjustment at 3.688.

Three minor QTLs were identified for the first harvest season, from August 2010 to January 2011 (**Table 5**, **Figure 6**), but barely above the threshold of significance (3.7) for the FAs linoleic and arachidic acid. The second harvest season, from May 2011 to November 2011, showed stronger QTLs among the FAs (**Table 5**, **Figure 7**), with the greatest peak from palmitic acid in LG04 (-log_10_ *p* 18.3, var. expl 16.9%). Stearic acid, oleic and arachidic acid had QTLs in LG04 with larger effect from oleic acid (-log_10_ *p* 11.2, var. expl 11.0%). Arachidic acid had QTLs with the strongest peak in LG09 (-log_10_ *p* 8.2, 8.3% var expl), and three minor QTLs in LG04 (-log_10_ *p* 6.6, 6.8% var. expl), 6 (-log_10_ *p* 4.6, var.expl 4.8%), and 8 (-log_10_ *p* 4.7, var expl 4.9%).

**Table 5:** QTL summary 1 -log_10_*p* away from peak for harvest seasons 1 and 2.

Harvest season	Trait	LG	-log_10_ *p*	% var expl	START	STOP	Peak marker	Range Mbps
1	C18:2	2	4.17	4.32	40,846,923	41,141,331	41,082,951	0.29
	C20:0	4	4.06	4.20	1,006,787	1,006,787	1,006,787	0
	C20:0	9	4.27	4.42	8,689,672	8,757,982	8,689,672	0.07
2	C16:0	4	18.32	16.93	311,794	1,307,427	311,794	1.00
	C18:1	4	11.16	11.03	1,006,787	1,006,787	1,006,787	0
	C20:0	9	8.18	8.31	6,064,842	8,372,974	6,064,842	2.31
	C20:0	4	6.58	6.77	19,637,361	26,233,319	19,637,361	6.60
	C18:0	4	5.15	5.33	615,809	712,489	709,649	0.10
	C20:0	8	4.74	4.92	2,227,678	5,284,396	3,274,675	3.06
	C20:0	6	4.63	4.80	23,105,784	24,526,606	24,366,215	1.42
	C18:2	5	4.56	4.73	29,143,790	31,845,564	29,405,116	2.70
	C18;1	6	4.51	4.67	10,137,698	16,515,632	13,900,060	6.38
	% Fat Content	5	4.05	4.20	36,125,454	36,125,454	36,125,454	0.00
	C18:0	5	3.88	4.01	4,070,263	4,670,177	4,606,155	0.60

This result indicates the stability of the arachidic acid QTL in LG09, which is present in both harvest seasons and the combined data sets with range of peak markers overlapping in the range of 6 to 9 Mbps. The difference in the significance (-log_10_ *p*) and percent of variation explained are possibly due to the different sample sizes between the harvest seasons, affecting statistical power of the QTL detection. In the case of palmitic acid, QTLs are present in the combined harvest seasons (LGs 1, 4, 7, 8, 9, and 10), and one QTL in LG04 in harvest season 2, and no presence of QTL in harvest season 1. Percent of total fat content is also a trait that has multiple QTLs in the combined seasons (LGs 2, 3, 4, 5, 6, and 9) with peak in LG02 (-log_10_ *p* 9.8, max var expl 9.8%) and one QTL in LG05 for the second harvest season -log_10_ *p* 4.1, %var expl 4.2%), with no presence of QTL in the first harvest season.

### Weather Correlations With FA Composition and Total Fat Content

Similar to described previously by Wintgens (1992) and by Lehrian et al. (1980 we observed a weak but significant positive correlation (*r* = 0.28, *p *< 0.001) between higher temperatures and palmitic acid content at the four-month moving average (lag4) (**Table 6**, **Supplementary Figure 4**). There was no difference between the lag3 and lag4 correlations with the traits, thus, we only present lag4 as representative. Increased temperatures for the month of harvest (lag0) and prior to harvest (lag1) were associated with increased stearic acid values. Oleic acid had a consistent, negative correlation with temperature (min, max, and average) for all lagged months, with a stronger correlation with maximum temperature observed at lag1 (*r* = −0.52, *p* < 0.001). Similarly, linoleic acid was also negatively correlated with temperature for most lags and was moderately associated with maximum temperature of the month prior to harvest (lag1, *r* = −0.41, *p* < 0.001). Total percentage of fat content also had a low, positive correlation with increased temperatures, particularly for the 6 months prior to harvest (lag6, *r* = 0.28, *p* < 0.001). In addition, temperature was not associated with percentage of fat content and palmitic acid for lag0 and lag1. The strongest linear relationships were observed between temperature and palmitic acid, with maximum temperatures at lag4 (*r* = 0.85, *p* < 0.001).

**Table 6:** Correlations between climate and traits.

Metric	lag	C16:0	C18:0	C18:1	C18:2	C20:0	% Fat Content
Avg. Temp	0	0.03	0.63*	−0.38	−0.41	0.45	0.12
Min. Temp	0	−0.04	0.54	−0.29	−0.30	0.39	0.20
Max. Temp	0	0.18	0.74**	−0.54	−0.61*	0.52	−0.08
Rain (mm)	0	0.12	−0.14	0.11	−0.19	−0.47	−0.38
Avg. Temp	1	0.39	0.69*	−0.65^*^	−0.68*	0.43	−0.11
Min. Temp	1	0.20	0.53	−0.43	−0.46	0.25	−0.04
Max. Temp	1	0.65*	0.80**	−0.88^***^	−0.90***	0.66*	−0.24
Rain (mm)	1	0.57	−0.23	−0.24	−0.17	0.01	0.23
Avg. Temp	4	0.75**	0.43	−0.78^**^	−0.62*	0.65*	0.36
Min. Temp	4	0.52	0.45	−0.63^*^	−0.52	0.54	0.38
Max. Temp	4	0.85**	0.17	−0.70^*^	−0.53	0.54	0.15
Rain (mm)	4	0.34	−0.08	−0.24	−0.02	0.32	0.38
Avg. Temp	6	0.57	0.26	−0.61^*^	−0.33	0.71**	0.63*
Min. Temp	6	0.5	0.38	−0.62^*^	−0.39	0.69*	0.60*
Max. Temp	6	0.45	−0.16	−0.29	−0.01	0.4	0.38
Rain (mm)	6	0.16	−0.60	0.19	0.43	−0.03	0.46

Pearson correlation coefficients between weather metrics, fat content and FAs. p Values used the “Holm” adjustment for multiple comparisons. Correlations are between the moving averages of the weather metrics and fatty acids. Lags are in terms of number of months prior to pod harvest, where E.g., Average temperature at lag = 0 is the average weather metrics for the current month vs the fat content and the FAs for the month of harvest, and lag = 4 is the average temperature of the 4 months prior to the current month of harvest. p Values are represented by asterisks (p < 0.0001 (***), p < 0.001 (**), p < 0.05 (*). p Values used the “Holm” adjustment for multiple comparisons.

Positive correlations between FAs and the amount of rainfall were significant for lags 1, 4, and 6, particularly for palmitic acid at lag1 (*r* = 0.27, *p* < 0.001), oleic acid at lag4 (*r* = −0.32, *p* < 0.001), and linoleic acid at lag6 (*r* = 0.22, *p* < 0.001). However, negative correlations were observed for stearic acid at lag6 (*r* = −0.20, *p* < 0.001) and arachidic acid at lag0 (*r* = −0.30, *p* < 0.001). Fat content had either negative or positive correlations with rainfall, depending on the lagged month. We observed that for the month of harvest, fat content had a negative correlation with rain (*r* = −0.20, *p* < 0.001) and a positive correlation when the average amount of rainfall is taken during the 6 months (pod formation) prior to harvest (*r* = 0.23, *p* < 0.001).

### Heritability

Most traits for which estimates of heritability have been reported (Ndoumbé et al., 2001; Lockwood et al., 2007; Cilas et al., 2010; Ofori et al., 2016; DuVal et al., 2017; Mustiga et al., 2018; Padi et al., 2017; Romero Navarro et al., 2017) show a range between 0.13 and 0.79. Heritabilities calculated for the FA content in this study (**Table 7**) also varied within that range (0.14–0.43). Interestingly, total fat content showed the lowest heritability value (0.14); this could be explained by the fact that it is a complex quantitative trait strongly influenced by both temperature and amount of rainfall for the longest periods (4 and 6 months before harvest) as shown in **Table 6**. Heritabilities of palmitic, stearic, and linoleic acids are highest (H^2^ = 0.43, 0.38, 0.35, respectively), and moderate heritabilities are reported for oleic and arachidic acids (H^2^ = 0.22, 0.26, respectively).

**Table 7:** Mean and standard deviation (SD) for the phenotypic fatty acid profiles and percentage of fat content in the MP01 population.

Trait	Mean	SD	H^2^ ${\hat{\sigma }}_{G}^{2}/{\hat{\sigma }}_{p}^{2}$	${\hat{\sigma }}_{y}^{2}/{\hat{\sigma }}_{p}^{2}$	${\hat{\sigma }}_{G*y}^{2}/{\hat{\sigma }}_{p}^{2}$	${\hat{\sigma }}_{\varepsilon }^{2}/{\hat{\sigma }}_{p}^{2}$
C16:0	28.7	1.80	0.43 ± 0.03	0.002 ± 0.003	0.038 ± 0.014	0.530 ± 0.022
C18:0	31.3	1.86	0.38 ± 0.04	0.059 ± 0.080	0.054 ± 0.015	0.512± 0.047
C18:1	34.8	1.32	0.22 ± 0.05	0.125 ± 0.156	0.052 ± 0.019	0.600 ± 0.108
C18:2	3.9	0.64	0.35 ± 0.03	0.013 ± 0.019	0.086 ± 0.018	0.547 ± 0.024
C20:0	0.9	0.10	0.26 ± 0.04	0.100 ± 0.128	0.009 ± 0.013	0.630 ± 0.092
% Fat Content	56.1	2.69	0.14 ± 0.03	0.103 ± 0.131	0.029 ± 0.017	0.731 ± 0.108

Proportion of variance components relative to the total phenotypic variance and respective standard errors are reported: Broad-sense heritability (H^2^, column 4), proportion of variance due to harvest season (column 5), proportion of variance due to interaction between harvest season and genetic variance (column 6), and proportion of residuals relative to the total phenotypic variance (column 7).

### Major QTL for Palmitic Acid Variation

A major QTL (defined here as explaining >15% of variation) on linkage group four explains 23.5% of the variation in palmitic acid levels, with a -log_10_ *p* score of 27.6 from the two combined harvest seasons (**Table 4**, **Figure 5**) and also present in the second harvest season (18.3 -log_10_ *p*, 16.9% var expl). The ordinary least squares regression of palmitic acid levels on the means of the four parental haplotype combinations (T1C1, T1C2, T1C2, and T2C2, with T representing “TSH 1188” and C representing “CCN 51”) for the peak marker confirms high significance (*p* << 0.0001). Tukey’s linear hypothesis shows the effect of T2 to be significantly higher (*p *< 0.001) than the genotypes containing T1, C1, and C2 in the analysis of the combined seasons. Hence, the T2 haplotype from “TSH 1188” has the greatest effect on increasing the level of palmitic acid (**Table 5**); the genotypes containing T2 have an average palmitic acid value of 29.4 %, which is, on average, 4.5% higher than T1 haplotype, and 2.4% and 2.0% higher than the C1 and C2 haplotypes, respectively (**Table 4**).

### Source of the Allele That Increases Palmitic Acid

**Figure 8:**
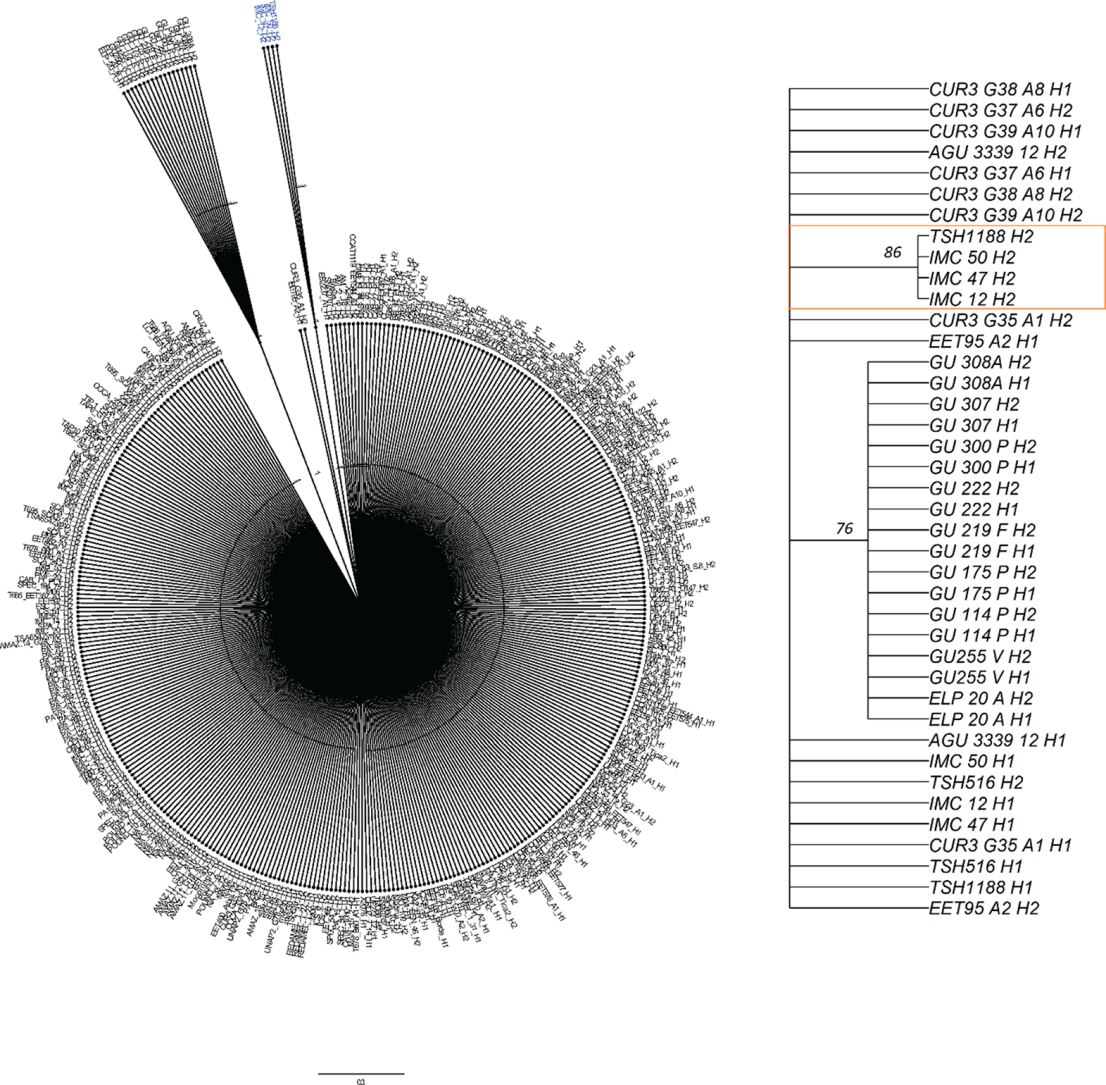
Neighbor joining tree for haplotypes from 200 diversity panel individuals. Zoomed in section for the palmitic haplotype from TSH1188 H2 haplotype and the cluster with Iquitos haplotypes with support values are on the right figure.

To identify the ancestry of the T2 allele that increases palmitic acid levels, an NJ tree of ancestral haplotypes was created from a diversity panel that includes “TSH 1188” and “CCN 51.” The T2 allele clustered with individuals that have a high percentage of the Iquitos genetic group (Motamayor et al., 2008) with a support value of 86% based on the 1000 bootstrap consensus tree (**Figure 8**). The phased alleles between T2 from “TSH 1188” and one of the phased alleles from “IMC 12,” “IMC 47,” and “IMC 50” are identical. “TSH 1188” is an admixed genotype, with 48% Iquitos, 16% Amelonado, 11% Contamana, 13% Marañon, 7% Criollo, 3% Guiana, and 2% Nacional ancestral groups (Romero Navarro et al., 2017). “IMC 12” and “IMC 50” have 100% and 93% Iquitos ancestry, respectively. “IMC 47” is more admixed, but also with highest proportion of Iquitos (58%), 21% Nacional, 11% Nanay, 6% Amelonado, and 5% Guiana. For reference, CCN51 is also admixed and has 49% Iquitos, 32% Amelonado, and 19% Criollo membership.

### Pollination Effects

Pollen effects on fat content and FA profile were evaluated from 76 trees pollinated with three sources of pollen: (i) open pollinated (OP), (ii) self-pollinated (SP), and (iii) clone “SIAL 70.”

Significant differences in the values of FAs were found between pollination types. Palmitic acid was significantly lower when “SIAL 70” was used as a pollen donor, stearic acid was highest with “SIAL 70,” oleic acid was lowest with SP, linoleic acid lowest with “SIAL 70,” and arachidic acid highest with SP. There was no evidence of pollen effects on total fat content (**Supplementary Table 6**).

## Discussion

### FA Variation in *T. cacao* Ancestral Groups

Preliminary chemical analyses of FA composition and total fat content of some of the progeny in the mapping population showed a wide range of variation, even though the parents displayed very similar FA compositions, with the exception that the maternal genotype, “TSH 1188” had slightly higher fat content. The high variation allowed us to identify various genomic regions associated with the tested traits. Total fat content in the State of Bahia in Brazil has been previously reported (Pires et al., 1998) from the germplasm collection at the Centro de Pesquisas do Cacau (CEPEC), with a mean of 53.2% from 490 accessions, ranging from 45.4% to 60.3% 

Despite the similarity of their phenotypes, the parents of the mapping population have different proportions and memberships to the ten previously identified ancestral groups (Motamayor et al., 2008). Thus, through recombination, the F1 progenies obtained combinations of those alleles that lead to a range of phenotypic values (transgressive segregation). The phenotypes of the progeny showed small variations for oleic acid and higher ranges of palmitic and stearic acid (**Table 2**). This could indicate that within the two parental genomes, there exists genetic variation for the major saturated fats in cocoa beans, but less so for genes affecting oleic acid.

### FA Content in *T. cacao* Relatives

Analysis of arachidic acid levels in the beans of other species of *Theobroma* showed similarly low levels (less than 2.1% of FAs) in *T. bicolor, T. microcarpum*, and *T. speciosum* (Carpenter, et al. 1994). In contrast, *T. grandiflorum*, which is cultivated for use in desserts and cosmetics in Brazil, has 9.8% to 12.1% arachidic acid, whereas the wild species *T. gileri, T. angustifolium*, *T. obovatum*, and *T. mammosum* had levels ranging from 8.6% to 13.4% (Carpenter et al., 1994). Bean FA composition from the sister genus Herrania was even more rich in arachidic acid, ranging from 16.3% to 20.1% of total FAs (Carpenter et al., 1994). Thus, while no significant differences have been found between FA profiles within the same *Theobroma* phylogeneic sections, significant differences have been reported between different *Theobroma* sections (Gilabert-Escrivá et al., 2002). The diversity of FA composition in *Theobroma* species can be valuable for breeding programs aiming for introgression of targeted FA profiles, fat content, and seed quality traits into *T. cacao.*

### QTL Identification

With a large QTL in chromosome 4, explaining more than 23% of the genotypic variation, palmitic acid can be selected against, given its identification of the source of the allele that increases palmitic acid originates from the maternal “TSH 1188” haplotype (T2). The three clones from our diversity group with the T2 haplotype form an Iquitos sub-group, “IMC 12” was previously reported as belonging to sub-group or sub-cluster IMCI in Motamayor et al. (2008). The FA profiles of cocoa beans and chocolates vary across geographic origins (Torres-Moreno et al., 2015). Environmental conditions affect FA in cocoa beans (Lehrian et al., 1980; McHenry and Fritz, 1987; Chaiseri and Dimick, 1989; Lipp and Anklam, 1998), but genetics may also play a role. Palmitic acid levels in selection of Criollo ancestral group cultivars ranged from 20% to 30% (Liendo et al., 1997), while the levels in the MP01 population ranged from 26% to 34%. The Iquitos allele that “TSH 1188” shares with the IMC genotypes is associated with the elevated palmitic acid levels, and is not present in the Ciollo ancestral group, implying that genetic backgrounds contribute to palmitic acid levels in beans.

Within linkage group 4 QTL, both the “B97-61/B2” and “Matina1-6” reference genomes have four copies of the *SAD/FAB2* stearoyl-ACP desaturase (Argout et al., 2011; Motamayor et al., 2013; Zhang et al., 2015). These are annotated as *TcSAD1*, *TcSAD2*, *TcSAD3*, and *TcSAD5*. In total, cacao has eight *SAD* homologues and Arabidopsis has seven (Argout et al., 2011). Examination of “CCN 51” and “TSH 1188” SNPs in the SAD genes and the surrounding regions shows extensive variation in the quantity of SNPs (**Supplementary Table 5**). Of particular interest are missense mutations in the coding region of “TSH 1188,” which could conceivably alter enzyme function. “TSH 1188” also has many more SNPs in *SAD2* and *SAD3*, especially in the downstream region, suggesting a high level of genetic variation.

Although arachidic acid comprised an average of 0.9% of bean FAs in the MP01 population, we identified three major QTLs for arachidic acid variation. The largest effect QTL was on chromosome 9 and explains 26.1% of the variation in arachidic acid (C20:0) levels with a -log_10_ *p* of 31.8 (**Table 4**). Close to this region, on chromosome 9, is *Thecc1EG037982* (Tc09cons_t010430.1 in Criollo v2 assembly), an orthologue of the Arabidopsis gene FAE1/KCS18, which encodes the 3-ketoacyl-CoA synthase 18. FAE1/KCS18 elongates saturated and monounsaturated FAs of 16 and 18 carbons (Ghanevati and Jaworski, 2002), and ketoacyl-CoA synthases play a role in arachidic acid biosynthesis in Arabidopsis (Millar and Kunst, 1997). Mapping in Arabidopsis found that a point mutation in *FAE1/KCS18* controlled variation in ratio between the long FAs (20 to 24 carbons, like arachidic acid) and short FAs (16 to 18 carbons, like palmitic and stearic acids) (Jasinski et al., 2012). For reference, there are 20 copies of *KCS* in cacao and 21 in Arabidopsis (Argout et al., 2011).

A minor QTL for variation in stearic acid was identified on linkage group 8 explaining 9.4% of variation with a -log_10_ *p* of 9.3. Within this region is *SAD7* (Thecc1EG035438). Like the other *SAD* genes, variation was detected between the two parents, with the “CCN 51” alleles identical to “Matina1-6,” and the “TSH 1188” containing multiple SNPs compared to “CCN 51.” (**Supplementary Table 5**).

*TcSAD1*, *TcSAD2*, *TcSAD3* are cacao orthologues that are closely related to the Arabidopsis *FAB2* gene (Zhang et al., 2015). The *fab2* mutant has increased levels of stearic acid and lower levels of oleic acid (Lightner et al., 1994; Kachroo et al., 2007) while knocking-down *SAD/FAB2* expression in rapeseed, maize, and Arabidopsis increased stearic acid levels in seeds (Knutzon et al., 1992; Du et al., 2016). Expression of Arabidopsis *FAB2* in *Escherichia coli* decreased the level of palmitic acid and resulted in the production of oleic acid that was otherwise absent in wild type *E. coli* (Cao et al., 2010). 

### Correlation Between FAs

From the analyses for the phenotypic correlation between traits, total fat content was positively associated with stearic acid and arachidic acid, and negatively correlated with palmitic, oleic, and linoleic acid (**Table 3**). Moreover, palmitic acid levels and the combined level of stearic, oleic, and linoleic acids were negatively correlated (**Figure 4**).

In addition, selection of genotypes in the 95th percentile for fat content (higher % fat) have significant differences in FA profile compared with genotypes that have low fat content (5th percentile). More specifically, the FA profile for high-fat genotypes contains high stearic (*p* = 0.012), lower oleic (*p* = 0.023), and linoleic (*p* = 0.042) FA profiles, and no differences in palmitic or arachidic acid values were found (**Supplementary Figure 3**). Although the high-fat genotypes are generally associated with higher saturated (C18:0) and lower unsaturated FAs (C18:1, C18:2), the selection of genotypes that are correlation breakers is feasible in breeding programs (**Supplementary Figure 2**). The strongest linear relationship observed was between palmitic and stearic acid (−0.74). Thus, based on the results from this mapping population, a clone with low palmitic acid, high stearic acid, and high cocoa butter content could be bred (**Supplementary Figure 2**). The relatively higher heritability for both palmitic acid (H^2^ = 0.43) and stearic acid (H^2^ = 0.38) may contribute to this process, even though heritability for fat content is low (H^2^ = 0.14) (**Supplementary Table 3**). The combination of low saturated and high unsaturated FA could be desirable for the food industry, as palmitic acid, but not stearic acid, has often been associated with negative cardiovascular health impacts (Mozaffarian et al., 2010; Zong et al., 2016).

This study also found a significant correlation between arachidic and palmitic acid (*r* = −0.37). This result could be related to the mechanism of the FA biosynthesis pathway regulation during seed development, where palmitic (C16:0) is converted to stearic (C18:0) by the KASII/FAB1 enzyme and C18:0 is converted to arachidic (C20:0) through elongases coded by FA elongation (FAE) gene. Species from the section *Glossopetalum* have been found to have significantly lower levels of palmitic and increased levels of arachidic than in *T. cacao* (Gilabert-Escrivá et al., 2002), suggesting the negative correlation between the two FAs. The negative correlation between C20:0 and C16:0 has also been identified in other plant species, such as in some peanut families and in wild safflower (Arslan, 2007; Wang et al., 2015).

### Effect of Climate Factors on FA Content

Complex quantitative traits are affected by several environmental factors, each having specific scales of impact on a crop, depending on the physiological stages of the crop and stage of seed development. Studies in developing embryos in cacao beans demonstrate that changes in FA composition occur between 95 and 115 days after pollination and during this period, linoleic and palmitic acid decrease relative to the total lipid amount, whereas stearic and oleic increase (Patel et al., 1994). Thus, changes in FA composition during embryo and seed development are a function of FA accumulation, growth rate of the embryo, and interaction with environmental factors. In our study, we found various levels of association between climatic factors including temperature and amount of rainfall during pod development with either increased or decreased FA levels. For each of the 12 harvest dates in the study, the means for FA and fat content were calculated and plotted against the average temperature and rain for different lags in time. For instance, at lag4, trees harvested in August 2011 would have been compared with the average temperature in April 2011 (3 months prior, during pod formation), and so on, for the 12 harvest dates, generating 12 data points for each lag comparison (**Table 6**, **Supplementary Figure 4**).

Arachidic acid had a weak positive correlation with temperature, particularly for the 6 months prior to pod harvest (lag6, *r* = 0.32, *p* < 0.001), also shown in Lehrian et al. (1980). Strong positive linear relationships were found between temperature and both palmitic and stearic acids, specifically for temperatures 4 to 6 months prior to harvest. Lower temperatures were strongly associated with increased levels of oleic and linoleic acid. This negative correlation for oleic acid was also reported by McHenry and Fritz (1987). These data are also in accordance with previous research, where lower temperatures were found to be associated with increased levels of linoleic acid (Canvin, 1965; Lehrian et al., 1980; Leathers and Scragg, 1989).

Interestingly, oleic acid is the first unsaturated FA that is generated from the saturated FAs produced by the FA synthase. In plants, oleic acid is produced via a stearoyl-[acp], an acyl-carrier protein-bound intermediate (Jaworski and Stumpf, 1974). Cheesbrough (1989) found that when culturing developing soybean seeds for 20 h in liquid media at 20°C, 25°C, or 35°C the total activity of stearoyl-ACP desaturase decreased twofold between 20°C and 25°C and sixfold between 20°C and 35°C cultures. This seems to be in line with lower temperatures and increased levels of the unsaturated FAs, oleic and linoleic acid during the lag4, especially when this lag4 corresponds with the colder months of the year, which also seems to coincide with the changes in FA composition that occur between 95 and 115 days after pollination (Patel et al., 1994).

The amount of rain 6 months prior to harvest was associated with reducing stearic acid levels and increasing both linoleic acid and total fat content. This may indicate that temperatures and rain tend to have more of an effect on FA during seed formation, before ripening of the pod. Previous studies on pod formation report that ripening occurs 140 days after pollination or 5 to 6 months from the time the flower is pollinated (Mckelvie, 1956; Glendinning, 1962).

### Pollen Donor Effects

The effect of pollen donor was significant in this study for FAs, but not for total fat content, even though previous research has shown a highly significant effect of pollen donors on fat content, in addition to interaction between parents (specific combining ability) (Pires et al., 1998). In our study, the pollen from the clone “SIAL 70” was associated with 5% and 8% decreased palmitic acid levels and increased stearic (4.3%) and oleic acid (5.4%) when compared with open or self-pollinated treatments, respectively (**Supplementary Table 6**). The effect of pollen source is known to impact the composition of FAs, affecting expression levels of the main desaturase genes involved (SAD and FAD), especially during rapid oil accumulation, typically during seed development (Xie et al., 2019).

## Conclusion

Our research provides further evidence that variation in cocoa bean fat composition has an environmental and genetic basis. Further research is needed to decompose the specific physiological events during bean formation that are the most affected by temperature and water availability influencing fat content and FA profile. Indeed, our studies were carried out without fertigation; it would be interesting to compare our results from studies implemented on fertigated plots. We have identified alleles with positive or negative effects on the fat profile (composition and content) through genetic mapping. Ultimately, the markers associated with fat content and FA composition reported here could be used to breed for the most desirable fat profile for chocolate producers and consumers. The low heritability of fat content and the costs associated with the analyses described suggest that including marker-assisted selection for these traits would be a cost-efficient approach to include in breeding programs to obtain various favorable traits. Furthermore, the use of appropriate pollen donors, further temperature monitoring during pod development months, and the identification of sites worldwide with the most adequate natural rainfall and temperature conditions could increase the probability of obtaining the desired FA composition in the cocoa beans.

## Author Contributions

GM analyzed the data and wrote the manuscript. JM and SR wrote the manuscript. CV-D, CB, EC, and ES collected data. JS, EC, LM, AD, and JJ analyzed data. SR, JCM, and JPM designed the trial. JPM managed collection of field data. JCM conceived the experiment and wrote the manuscript.

## Conflict of Interest

Author(s) Guiliana M. Mustiga, Joe Morrissey, Joseph Conrad Stack, Ashley DuVal, Stefan Royaert, Carolina Bizzotto, Cristiano Villela-Dias, Ed Seguine, Jean Philippe Marelli and Juan Carlos Motamayor are/were employed by company(ies) Mars Chocolate, Mars Center for Cocoa Science and Guittard Chocolate Co at the time of the research or the writing of this paper. The remaining authors declare that the research was conducted in the absence of any commercial or financial relationships that could be construed as a potential conflict of interest.

## Supplementary Material

Supplementary Table 1 Climate data for each of the 12 months sampled. The minimum, maximum, and average temperatures (Cº) (Min., Max., Temp) are reported for each of the harvest months and season with the amount of rain fall (mm) and the FA averages.

Supplementary Table 2 Fatty acid composition of beans of the MP01 parents. Average percent FA composition of beans from MP01 parents, and percent total fat. Descriptive statistics for parental fatty acid phenotypic values for the period evaluated for the parents from July 2010-November 2011. Total number of observations (*n*) are *n* = 22 and *n* = 28 for “TSH 1188” and “CCN 51,” respectively. *p* is the *p* value from the t-test for comparing the means of the two parents.

Supplementary Table 3 Coordinates of *FAB2/SAD* candidate genes from palmitic acid major QTL.

Supplementary Table 4 Mapping of *SAD* gene models in the identified FA QTLs. QTL mapping results for the markers near the *FAB2/SAD* candidate genes with the Matina v2 coordinates.

Supplementary Table 5 SNPs variations relative to Matina1-6 reference genome (Motamayor et al., 2013). Upstream and downstream refer to 200 bp region from UTR.

Supplementary Table 6 ANOVA with Mean, SD, F-statistic, Mean Square and *p* value for pollination effect from 70 genotypes of the MP01 population.

Supplementary Figure 1 PCA for the FA traits and total fat content from average of both harvest seasons, with *n* = 420 genotypes.

Supplementary Figure 2 Biplot of 420 genotypes in MP01. Highlighted in red are trees with higher fat (95^th^ percentile, % fat >= 58.4) and lower palmitic fatty acid (10^th^ percentile, C16:0 <= 27).

Supplementary Figure 3 Boxplot of fatty acid profiles from individuals within the 5^th^ and 95^th^ percentiles for total fat content. *N*=21 for both groups. P-value is the T-test for comparison of means between the low fat (5^th^ percentile) and higher fat (95^th^ percentile) genotypes.

Supplementary Figure 4 Temperature, fatty acids and % fat content plotted against the weather metrics (Average, minimum, and maximum temperatures) at specific lags for which the correlations were most significant based on (Table 7). Each point on the plot (12 points) represents the average FA and fat content vs the temperature lag for the month of harvest.
