# Supplementary figures and images for: Identification of Climate and Genetic Factors That Control Fat Content and Fatty Acid Composition of Theobroma cacao L. Beans
Source: Front Plant Sci. 2019 Oct 14;10:1159. doi: 10.3389/fpls.2019.01159 (PMC6802002; doi:10.3389/fpls.2019.01159)

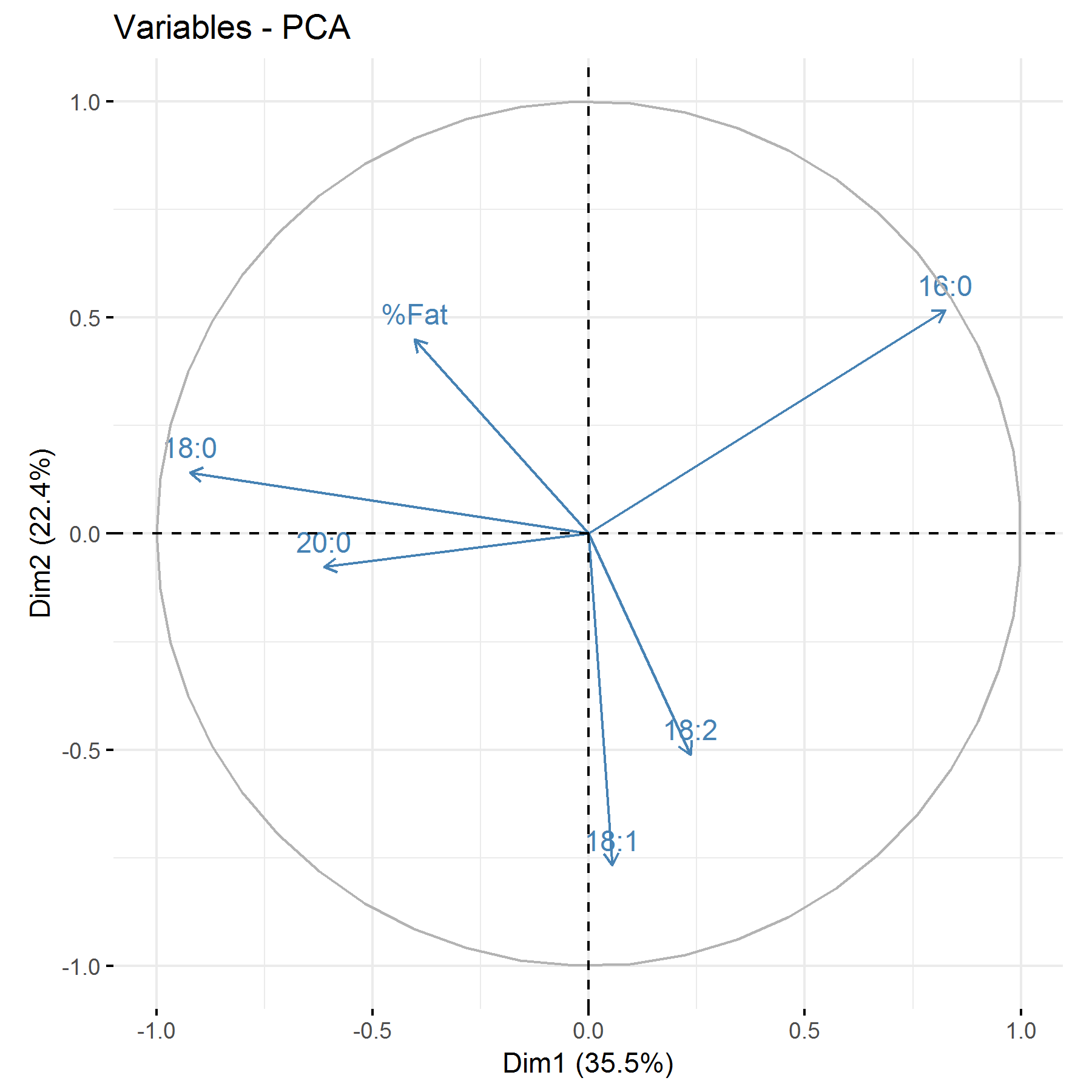

Supplement: Supplementary Figure 1 — PCA for the FA traits and total fat content from average of both harvest seasons, with n = 420 genotypes. [file Image_1.tiff]

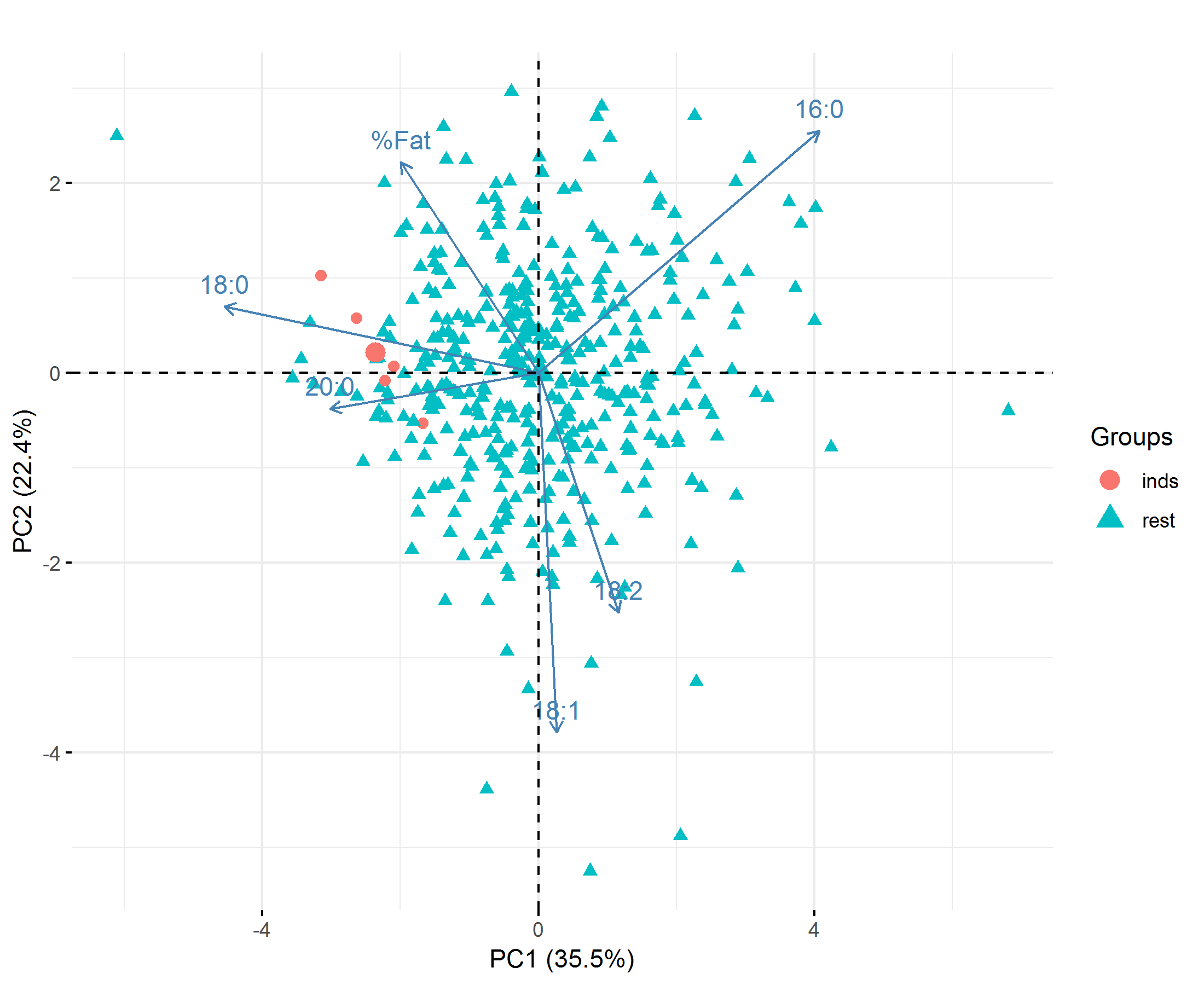

Supplement: Supplementary Figure 2 — Biplot of 420 genotypes in MP01. Highlighted in red are trees with higher fat (95th percentile, % fat >= 58.4) and lower palmitic fatty acid (10th percentile, C16:0 <= 27). [file Image_2.tiff]

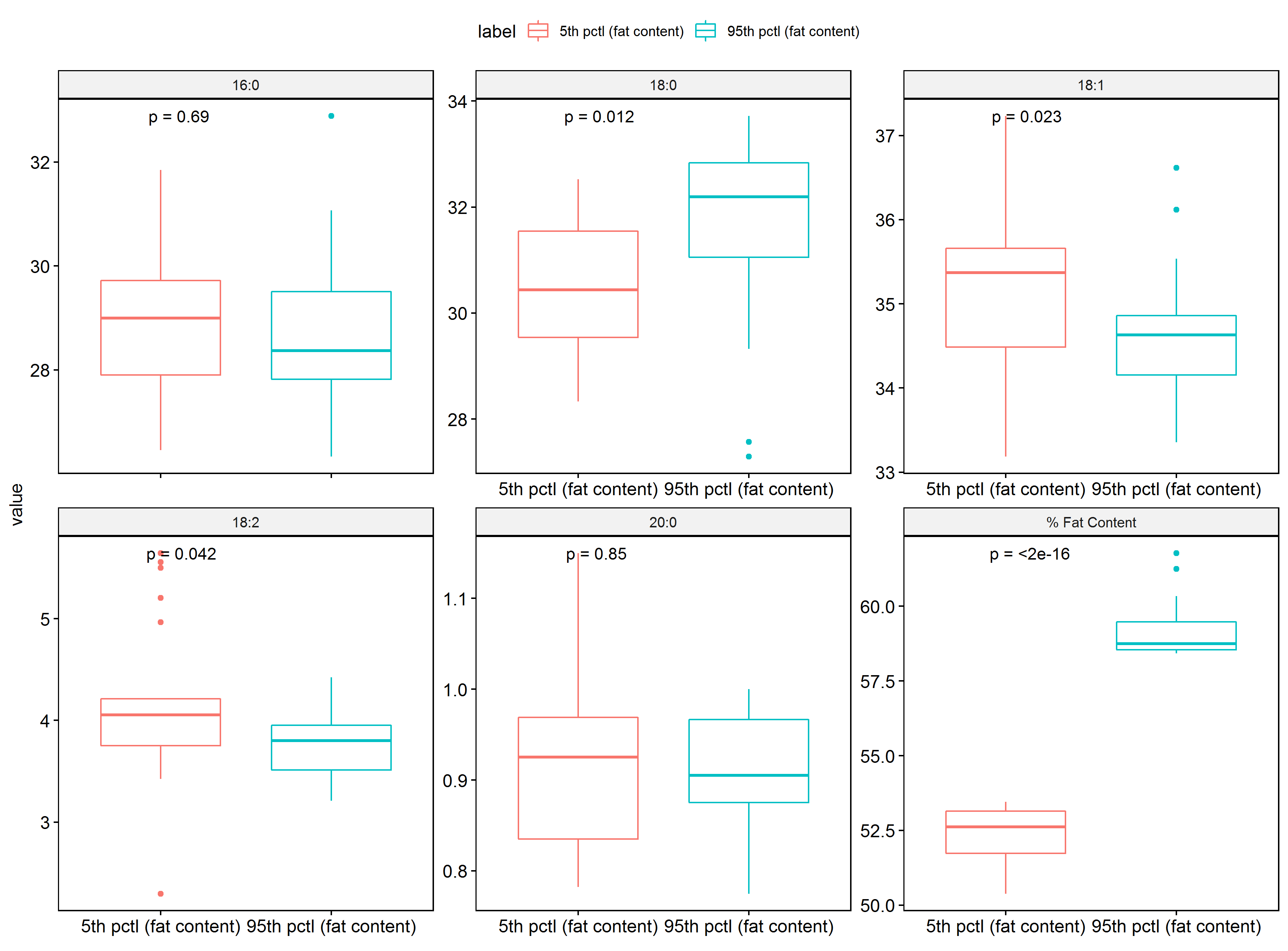

Supplement: Supplementary Figure 3 — Boxplot of fatty acid profiles from individuals within the 5th and 95th percentiles for total fat content. N=21 for both groups. P-value is the T-test for comparison of means between the low fat (5th percentile) and higher fat (95th percentile) genotypes. [file Image_3.tiff]

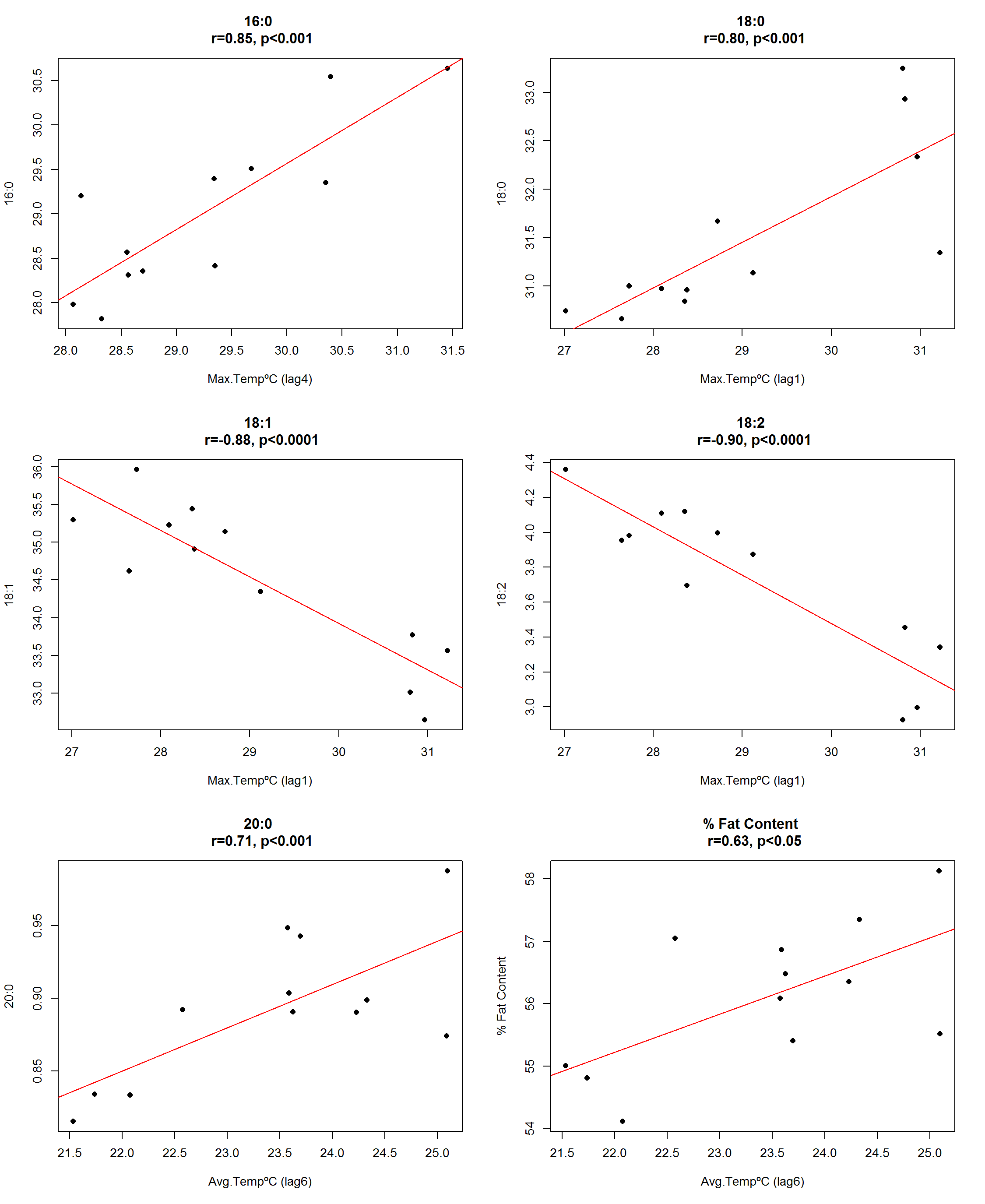

Supplement: Supplementary Figure 4 — Temperature, fatty acids and % fat content plotted against the weather metrics (Average, minimum, and maximum temperatures) at specific lags for which the correlations were most significant based on (Table 7). Each point on the plot (12 points) represents the average FA and fat content vs the temperature lag for the month of harvest. [file Image_4.tiff]
